# Role of endothelial *Dlc1* in embryonic vascular development and adult bone marrow hematopoiesis

**DOI:** 10.1038/s41375-026-03013-1

**Published:** 2026-06-25

**Authors:** Jing-Xin Feng, Marian E. Durkin, Mei-Ting Yang, Lili Li, Ross Lake, Caiyi Li, Ferenc Livak, Lino Tessarollo, Michael Kruhlak, Douglas R. Lowy, Giovanna Tosato

**Affiliations:** 1 Laboratory of Cellular Oncology, Center for Cancer Research, National Cancer Institute (NCI), National Institutes of Health, Bethesda, MD USA; 2 Experimental Immunology Branch, Center for Cancer Research (CCR), National Cancer Institute (NCI), National Institutes of Health, Bethesda, MD USA; 3 LCBG Microscopy Core, Laboratory of Cancer Biology and Genetics, CCR/NCI/NIH, Bethesda, MD USA; 4 Flow Cytometry Core, Laboratory of Genome Integrity, Center for Cancer Research, National Cancer Institute (NCI), National Institutes of Health, Bethesda, MD USA; 5 Flow Cytometry Facility, Janelia Research Campus, Howard Hughes Medical Institute, Ashburn, VA USA; 6 Neural Development Section, Mouse Cancer Genetics Program, Center for Cancer Research, National Cancer Institute (NCI), NIH, Frederick, MD USA; 7 Center for Cancer Research Microscopy Core, Laboratory of Cancer Biology and Genetics, NCI, NIH, Bethesda, MD USA

**Keywords:** Haematopoietic stem cells, Myelopoiesis

## Abstract

Bone marrow endothelial cells (ECs) form specialized vascular niches that support hematopoietic stem and progenitor cells (HSPC), yet the molecular regulators of this function remain incompletely defined. Here, we identify the Rho GTPase-activating protein Dlc1 as an essential regulator of developmental vasculogenesis, and adult bone marrow vascular niche integrity. Endothelial-specific deletion of *Dlc1* caused embryonic lethality with severe vascular defects. In adult mice, inducible EC-specific *Dlc1* deletion disrupted the bone marrow vascular architecture, associated with a significant reduction of multipotent hematopoietic progenitors and myeloid-lineage cells. Single-cell RNA sequencing revealed transcriptional reprogramming of *Dlc1*-deficient ECs, with the emergence of cell subsets displaying altered transcriptional profiles and disrupted expression of key niche signals, including *Kitl*, *Cxcl12*, and *Pdgfb*. Ligand-receptor interactome analysis demonstrated impaired EC-to-HSPC communication, and the hematopoietic cells exhibited transcriptional features of metabolic stress and reduced biosynthetic activity. These findings position *Dlc1* as a central regulator of developmental vasculogenesis, adult vascular architecture in the bone marrow, and EC-derived hematopoietic cell support. This work uncovers a previously unrecognized role for *Dlc1* in coupling vessel function to hematopoietic cell output, with implications for understanding bone marrow failure syndromes and targeting endothelial dysfunction in hematologic diseases.

## Introduction

Deleted in Liver Cancer 1 (*Dlc1*) encodes an evolutionarily conserved Rho GTPase–activating protein (RhoGAP), first identified as a tumor suppressor frequently deleted in hepatocellular carcinoma, and later linked to suppression of other cancer types, including multiple myeloma and acute myelogenous leukemia [1, 2]. *Dlc1* negatively regulates RhoA signaling and participates in cytoskeletal cell organization [3–7]. In endothelial cells (ECs), *Dlc1* inhibits VEGF-driven angiogenesis and modulates EC proliferation and migration [7, 8]. Constitutive *Dlc1* knockout in mice results in embryonic lethality around E10.5, with severe neural, cardiac, and vascular defects [9]. However, whether endothelial *Dlc1* contributes to these phenotypes remains unclear. Furthermore, early lethality in the global knockout has precluded investigation of *Dlc1* function in adult tissues.

ECs in the bone marrow play essential roles beyond controlling aspects of blood vessel function, as they form vascular “niches” that regulate hematopoietic stem and progenitor cells (HSPCs) through direct contact and angiocrine signaling [10]. Whether *Dlc1* contributes to these niche-supportive functions is unknown.

To address these questions, we generated mice with EC-specific *Dlc1* deletion using both constitutive (Cdh5-Cre) and inducible (Cdh5-Cre^ERT2^) systems. We demonstrate that EC-specific *Dlc1* deletion is embryonically lethal, revealing a critical role of *Dlc1* in early vascular development. In adult mice, loss of endothelial *Dlc1* disrupts the bone marrow vasculature and impairs hematopoietic cell support. These findings identify *Dlc1* as an essential regulator of endothelial cell functions during development and adulthood.

## Materials and Methods

### Mice and tamoxifen treatment

No human subjects were involved in this study. All animal experiments were approved by the National Cancer Institute (Bethesda campus) Intramural Animal Care and Use Committee (ACUC) and were performed in accordance with the relevant guidelines and regulations.

*Dlc1*^fl^ mice (exon 5 floxed) were generated as detailed in the Supplementary information. *Cdh5-Cre(7Mlia)* and tamoxifen-inducible *Cdh5-Cre*^*ERT2*^ lines were used for EC-specific recombination. Tamoxifen (100 mg/kg) was administered by oral gavage with progesterone (50 mg/kg) to pregnant females for embryonic deletion, or alone to 3–4-week-old mice (100 mg/kg for 3 consecutive days) for adult deletion.

### Embryo and tissue analyses

Embryos were collected at E11.5 or E13.5, genotyped, and examined under a stereomicroscope. Tissues were fixed in 4% paraformaldehyde. Paraffin sections (5 µm) were stained with hematoxylin/eosin. Cryosections (10 µm) were stained for CD34 (Abcam, #ab81289), with DAPI counterstain. For three-dimensional imaging, embryos and long bones were cleared using the EZ Clear protocol and imaged on Zeiss LSM 880 or Nikon SoRa confocal microscopes.

### Flow cytometry and cell sorting

Bone marrow (BM) cells were isolated by flushing or crushing femurs and tibiae, lysing red cells (ACK buffer) and blocking Fc receptors. For HSPCs analysis, lineage⁺ cells were depleted (BioLegend #480004). ECs were enriched as CD45⁻CD31⁺ populations; HSPCs as Lin⁻Sca-1⁺c-Kit⁺ (LSK). Data were acquired on BD FACSymphony or Sony ID7000 instruments and analyzed with FlowJo v10.8.

### Bone marrow EC culture and *Dlc1* silencing

The murine BM EC line BMEC-Akt1 [11] was maintained in DMEM/F-12 medium with 20% FBS and endothelial cell supplements (Millipore Sigma # E2759). Lentiviral shRNAs targeting *Dlc1* (Sigma TRCN series) or control vectors were packaged in HEK293T cells, concentrated by ultracentrifugation, and used to infect BMECs. Stable cell lines were selected with 3 µg/mL puromycin.

### Ex vivo ECs-HSPC co-culture

Sorted LSK cells from *mTmG* mice were seeded onto confluent BMEC monolayers and cultured in StemSpan medium (STEMCELL Technologies # 09605) with mouse SCF (PeproTech, #250-03) [12] and supplements for 4 weeks. Non-adherent cells were collected weekly for quantification of tdTomato⁺ cells. At the end of culture, adherent and non-adherent cells were quantified as tdTomato^–^ (endothelial) and tdTomato^+^ (hematopoietic) to assess hematopoietic expansion and EC viability.

### Single-cell RNA-seq

CD45⁻CD31⁺ZsGreen⁺ ECs and LSKs were sorted from tamoxifen-treated *Cdh5-Cre*^*ERT2*^*/ZsGreen/Dlc1*^*fl/fl*^ and control mice (*Cdh5-Cre*^*ERT2*^*/ZsGreen/Dlc1*^*fl/+*^) and processed using the 10x Genomics Chromium GEM-X Single Cell 3′ v4 system. Libraries were sequenced on an Illumina NextSeq 2000 and analyzed with Cell Ranger v9.0, Scanpy, and CellChat. Cell types were annotated with CellTypist [13]. Raw data of single-cell RNA-seq are available at the Sequence Read Archive (SRA, PRJNA1355203).

### Statistics

Data are presented as means ± SD. Two-tailed unpaired Student’s *t* tests were used for pairwise comparisons (*P* < 0.05 considered significant). χ² tests were used for genotype or phenotype frequencies.

### Other methods

Additional experimental details, including cell adhesion assays, cytokine profiling, genotyping, quantitative image analysis, and computational methods, are provided in the Supplementary information.

## Results

### Endothelial-specific *Dlc1* deletion causes vascular defects and embryonic lethality

**Fig. 1:**
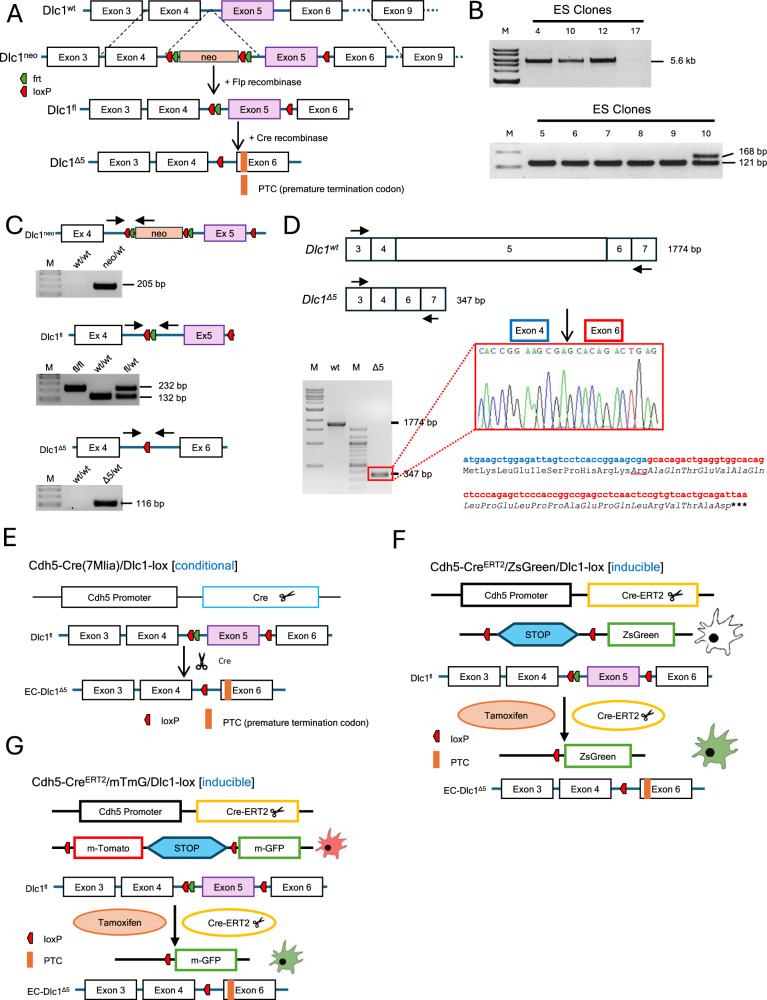
Generation of the conditional *Dlc1* exon 5 deletion allele. **A** Schematic representation of exons 3 to 9 of the mouse *Dlc1* gene (transcript variant 2) and targeting strategy. Homologous recombination introduced a loxP/frt-flanked neomycin cassette (neo) into intron 4 and a single loxP site into intron 5 of the wild-type allele (*Dlc1*^wt^). Flpe recombinase excised the neo cassette to generate the floxed allele (Dlc1fl). Cre recombinase deletes exon 5, producing a null allele (*Dlc1*^Δ5^) with a premature termination codon (PTC) in exon 6. Dotted lines indicate 5′ and 3′ homology arms used in the targeting vector. **B** PCR screening of genomic DNA from drug-resistant embryonic stem cell (ES) clones. Top: long-range PCR using primers in intron 3 and the neo cassette amplified a 5.6-kb fragment in correctly targeted clones 4, 10, and 12. Bottom: primers flanking the single loxP site in intron 5 amplified a 168-bp product in clone 10 in addition to the 121-bp wild-type band. **C** Genotyping of mice carrying *Dlc1* mutant alleles. Top: primers in intron 4 and in the neo cassette (pointed by the arrows) amplified a 205-bp product in *Dlc1*^neo^ mice. Middle: primers flanking the loxP/frt site amplified a 132-bp band from the *Dlc1*^wt^ allele and a 232-bp band from the *Dlc1*^fl^ allele. Bottom: primers in intron 4 and in intron 5 amplified a 116-bp band after Cre-mediated deletion of exon 5 (*Dlc1*^Δ5^). M, molecular size marker. **D** Validation of the *Dlc1*^Δ5^ transcript. Top: schematic of exons 3 to 7 in wild-type (WT) and exon 5-deleted transcripts. RT-PCR of heart tissue cDNA from *Dlc1*^wt/wt^ and *Dlc1*^Δ5/wt^ mice using exon 3 and exon 7 primers yielded a 1774-bp product from the wild-type transcript and a 347-bp product lacking exon 5. Bottom left: agarose gel of purified RT-PCR products. Bottom right: Sanger sequencing confirmed that exon 4 is spliced directly to exon 6 in the mutant transcript. Exon 5 deletion causes a frameshift after Arg85 of the 1092-amino acid DNA protein, introducing a premature stop codon (***). M, molecular size marker. **E** Schematic of approach to deletion of Dlc1 in endothelial cells by crossing Cdh5-Cre(7Mlia) mice with *Dlc1*^fl^ mice. Deletion of exon 5 produces the *Dlc1*^Δ5^ allele. Schematic of approach for the inducible deletion of Dlc1 in endothelial cells from *Cdh5-Cre*^*ERT2*^ / *Dlc1*^fl/fl^ mice carrying *ZsGreen* (**F**) or *mTmG* (**G**) Cre-reporter alleles. Tamoxifen administration at E8.5–E10.5 activates Cre^ERT2^-mediated recombination, resulting in exon 5 deletion of *Dlc1*. In the *ZsGreen* reporter mouse, fluorescence is absent until Cre activation induces ZsGreen expression; in the *mTmG* reporter mouse, tamoxifen-induced recombination switches fluorescence from red (tdTomato) to green (GFP).

To assess the role of *Dlc1* in ECs, we generated mouse lines allowing conditional and inducible deletion of *Dlc1* in ECs (Fig. 1A–G). A floxed *Dlc1* allele (*Dlc1*^fl^) was constructed by inserting a loxP/frt-flanked neomycin resistance (neo) cassette in intron 4 and a single loxP site in intron 5 of the *Dlc1* gene (Fig. 1A). After Flp-mediated removal of the neo cassette, Cre recombinase–mediated deletion of exon 5 introduced a premature termination codon (PTC) in exon 6, resulting in a truncated DLC1 protein lacking the catalytic RhoGAP domain and binding sites for tensin, talin, and caveolin [14] (Fig. 1B–D). To validate our floxed allele, *Dlc1*^fl^ mice were crossed with a “Cre-deleter” strain [15] to produce a global deletion of exon 5 (*Dlc1*^*Δ5*^). RT-PCR confirmed exon 4-6 splicing, and no viable *Dlc1*^Δ5/Δ5^ pups were recovered. Homozygous embryos exhibited mid-gestation lethality and developmental defects, consistent with the phenotype of the constitutive *Dlc1* knockout [9].

To evaluate the specific contribution of endothelial *Dlc1*, we crossed *Dlc1*^fl^ mice with the endothelial-specific *Cdh5-Cre(7Mlia)* line [16, 17] (Fig. 1E). Among 1317 pups, no live-born *Cdh5-Cre(7Mlia)*^+^/*Dlc1*^fl/fl^ were observed (χ² test, *p* < 1 × 10^-30^). Additionally, no homozygous embryos were recovered at E6.5 (0/21; χ² test, *p* < 0.05), indicating lethality around the onset time of yolk-sac vasculogenesis [18]. These findings establish endothelial *Dlc1* as essential for early embryogenesis.

**Fig. 2:**
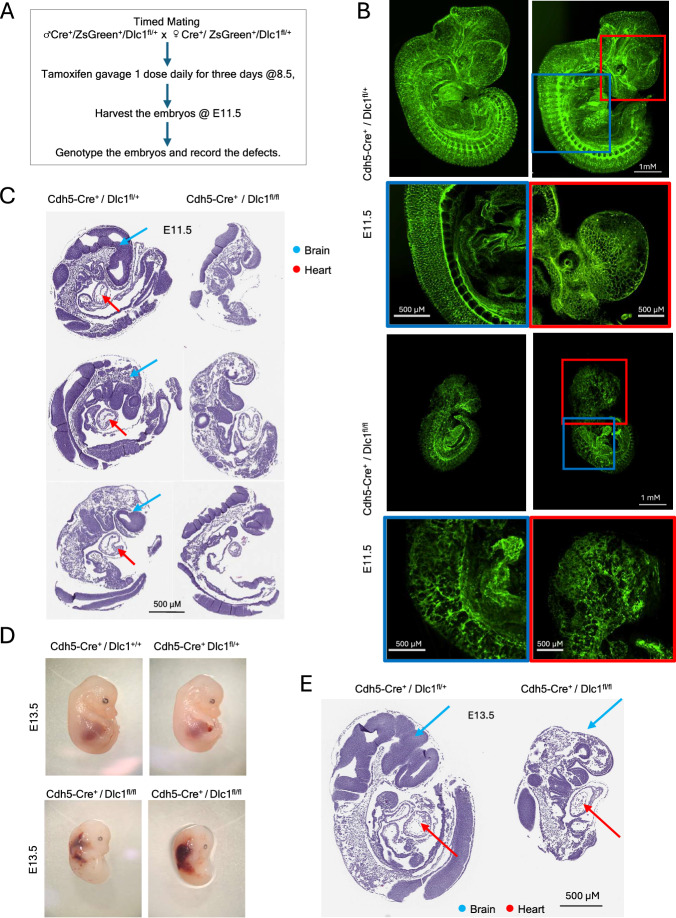
Endothelial-specific deletion of Dlc1 causes embryonic vascular defects and lethality. **A** Experimental design for the inducible endothelial-specific deletion of *Dlc1* in E11.5 embryos. Timed mating of Cre^+^ / *Dlc1*^fl/+^ males and females, followed by tamoxifen oral gavage (one dose/day for 3 consecutive days starting at E8.5); embryos were harvested at E11.5, and genotyped. **B** Whole-mount confocal imaging of E11.5 embryos with the indicated genotypes. After harvest, embryos were optically cleared using the EZView method and imaged by confocal microscopy from the surface to a depth of 500μm. 3D reconstructions were performed using Imaris software. Cdh5-Cre^+^/*Dlc1*^fl/fl^ embryos exhibit reduced size and disrupted vascular networks compared to control littermates. Green fluorescence is from ZsGreen. **C** H&E staining of E11.5 embryo sections. Compared to Cre^+^/*Dlc1*^fl/+^ controls, Cre^+^/*Dlc1*^fl/fl^ embryos exhibit reduced size and disrupted tissue architecture. **D** Representative brightfield images of E13.5 embryos. *Cdh5-Cre*^ERT2+^/*Dlc1*^fl/fl^ embryos exhibit gross malformations and hemorrhaging. **E** H&E staining of E13.5 embryo sections. Compared to Cre^+^/*Dlc1*^fl/+^ controls, Cre^+^/*Dlc1*^fl/fl^ embryos exhibit a spectrum of defects, including brain growth retardation, and small embryonic size.

Because the early lethality caused by constitutive endothelial deletion of *Dlc1* precluded tissue analysis, we adopted an inducible *Cdh5-Cre*^*ERT2*^ mouse model to temporally delete *Dlc1* in ECs, using ZsGreen or mTmG fluorescence reporters (Fig. 1F, G) [19, 20]. Pregnant females received tamoxifen from E8.5 to E10.5, and embryos were examined at E11.5 (Fig. 2A). While all *Dlc1*^+/+^ (18/18) and *Dlc1*^fl/+^ (54/54) embryos appeared normal and had a heartbeat, only 8/21 *Dlc1*^fl/fl^ embryos had a heartbeat (χ² test, *p* = 6.2×10^–5^). Whole-mount confocal imaging of E11.5 embryos revealed that *Dlc1*^fl/fl^ embryos were smaller, and displayed brain malformations and a disrupted vasculature in the brain and spine (Fig. 2B, C, and Supplementary Fig. S1A, C).

Since the E11.5 embryos induced with tamoxifen at E8.5 displayed widespread structural defects, we next induced *Dlc1* deletion at E10.5 and examined embryos at E13.5. All *Dlc1*^+/+^ (12/12) and nearly all *Dlc1*^fl/+^ embryos (29/30) were viable and had beating hearts, whereas only 1/14 *Dlc1*^fl/fl^ embryos had a heartbeat (χ² test, *p* = 1.5e−8). The *Dlc1*^fl/fl^ embryos were markedly smaller and displayed evidence of hemorrhaging (Fig. 2D), with histological evidence of global defects, including brain hypoplasia and abnormal cardiac chambers (Fig. 2E). Additionally, CD34 immunostaining and fluorescence imaging confirmed reduced and broadly compromised vascularization in the mutant embryos (Supplementary Fig. S1D, E).

These results demonstrate that endothelial *Dlc1* is required for physiologic vasculogenesis and embryonic development.

### Endothelial *Dlc1* deletion disrupts the adult bone marrow vasculature

**Fig. 3:**
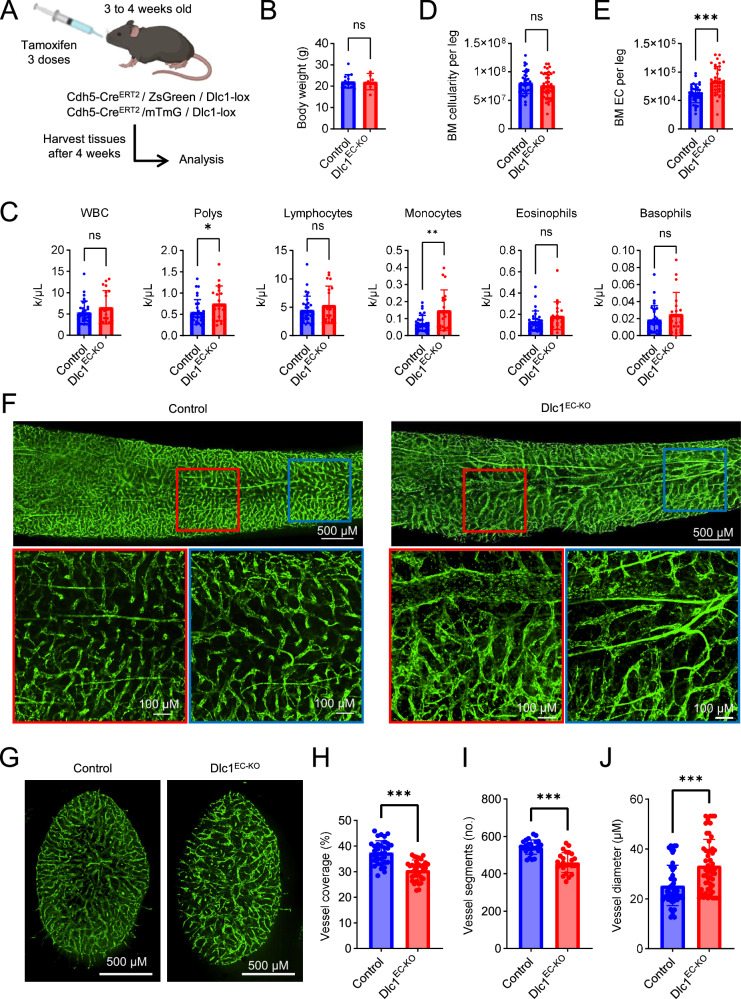
Dlc1 deletion in adult endothelial cells disrupts the bone marrow vasculature. **A** Illustration of experimental design. *Cdh5-Cre*^*ERT2*^ mice carrying mTmG or ZsGreen and *Dlc1*^*fl*^ alleles were treated with three doses of tamoxifen at 3 to 4 weeks of age. Bone marrow was harvested four weeks later for flow cytometry and confocal imaging. **B** Body weight of control (*n* = 15) and *Dlc1*^*EC-KO*^ (*n* = 6) mice. ns: not significantly different by unpaired two-tailed Student’s t-test. Each dot represents one mouse. The error bar represents the standard deviation (SD). **C** Complete blood count (CBC) analysis of peripheral blood from control and *Dlc1*^*EC-KO*^ mice. *P < 0.05, **P < 0.01, unpaired two-tailed Student’s *t*-test. Each dot represents result from one mouse (Control, *n* = 31; *Dlc1*^EC-KO^, *n* = 19). **D** Bone marrow cellularity in control (*n* = 37) and *Dlc1*^*EC-KO*^ (*n* = 38) mice. The results reflect the number of nucleated cells/leg (femur + tibia). **E** The number of bone marrow endothelial cells (BM EC) per leg is significantly increased in the *Dlc1*^*EC-KO*^ group compared to control; *** *P* < 0.001, unpaired two-tailed Student’s t-test. (Control, *n* = 31; *Dlc1*^EC-KO^, *n* = 19). **F** Representative sagittal sections images of whole-mounted bone marrow cleared using the EZView method. Images show a single optical section (matched 2.8 μm sections in control and *DLC1*^EC-KO^ bone marrow). The red and blue rectangles (upper panels) limit areas magnified in the corresponding lower panels. Compared to control mice, *Dlc1*^*EC-KO*^ mice display a visibly altered vascular network with fewer and more dilated vessels. ZsGreen fluorescence identifies endothelial cells. **G** Representative confocal images of bone marrow sectioned horizontally through the mid diaphysis from control and *Dlc1*^*EC-KO*^ mice, showing the vascular architecture. Quantification of vessel coverage (**H**), vessel segments (**I**), and vessel diameter (**J**) shows a significant reduction in the *Dlc1*^*EC-KO*^ compared to control mice; *** *P* < 0.001, unpaired two-tailed Student’s *t*-test. Measurements were taken on confocal images acquired at 10μm distance from each other, across a 400 μm z-stack from whole-mount marrow samples. The dots represent individual image quantifications. **H**, *n* = 25; **I**, *n* = 35; **J**, *n* = 50.

To assess the role of *Dlc1* in post-natal vessel homeostasis, 3- to 4-week-old *Cdh5-Cre*^*ERT2*^/*ZsGreen/Dlc1*^fl/fl^ mice were treated with tamoxifen and analyzed 4 weeks later (Fig. 3A). Immunofluorescence confirmed robust Cre-reporter-induced labeling of ECs following tamoxifen induction (Supplementary Fig. S2A). Efficient deletion of *Dlc1* exon 5 in bone marrow (BM) ECs was validated by PCR on genomic DNA from sorted fluorescent CD31^+^ ECs from the BM of *Cdh5-Cre*^*ERT2*^*/mTmG/Dlc1* mice (Supplementary Fig. S2C).

The mice with *Dlc1*-depleted EC (*Dlc1*^EC-KO^) appeared healthy with normal body weight (Fig. 3B). Also, complete blood cell counts in these *Dlc1*^EC-KO^ mice were similar to controls, except for modest increases in circulating polymorphonuclear cells and monocytes (Fig. 3C, Supplementary Fig. S2E). A preliminary histological evaluation of the main organs (heart, lung, intestine, spleen, kidney, and liver) revealed no gross abnormalities in *Dlc1*^EC-KO^ mice (Supplementary Fig. S3A), suggesting no apparent pathology under steady-state conditions.

We next examined the BM. Cellularity in the BM of *Dlc1*^EC-KO^ mice was overall normal (Fig. 3D). However, the number of BM ECs was significantly increased in *Dlc1*^EC-KO^ mice (Fig. 3E, *P* < 0.001), suggesting altered EC turnover. Whole-mount imaging of optically cleared femoral BM revealed vascular abnormalities in *Dlc1*^EC-KO^ mice: vessel density and branching were markedly reduced, and many vessels were abnormally dilated (Fig. 3F–J), all confirmed by quantitative analyses. Together, these results demonstrate that *Dlc1* is required to maintain a normal vascular architecture in the adult mouse BM.

### Endothelial *Dlc1* deletion in adult mice disrupts bone marrow hematopoiesis

**Fig. 4:**
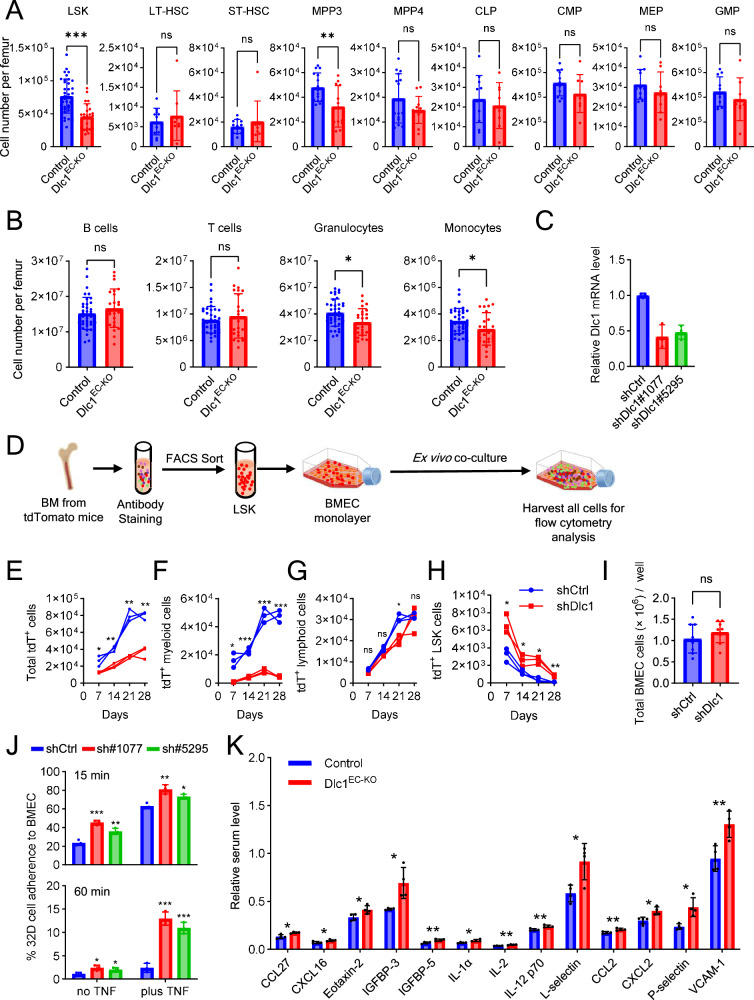
Endothelial-specific Dlc1 deletion in the adult mouse alters bone marrow hematopoiesis in vivo and ex vivo. **A** Analysis of bone marrow hematopoietic stem and progenitor cell subsets. *Dlc1*^*EC-KO*^ mice exhibited a significant decrease in the number of LSK (Lin⁻Sca1⁺c-Kit⁺) and MPP3 (CD135^-^CD150^-^CD48^+^ LSK); LSK: control *n* = 37, *Dlc1*^*EC-KO*^ mice *n* = 24; MMP3: control *n* = 15, *Dlc1*^*EC-KO*^ *n* = 12 mice. The number of LT-HSCs (long-term HSCs, Lin^-^ Sca-1^+^ cKit^+^ CD34^-^ CD135^-^ CD150^+^ CD48^-^), ST-HSCs (short-term HSCs, Lin⁻ Sca-1⁺ c-Kit⁺ CD34⁺ CD135⁻ CD150⁺ CD48⁻), MPP4 (CD135^+^CD150^-^ LSK), and CLPs (common lymphoid progenitors, Lin⁻ Sca-1^low^ c-Kit^low^ CD135⁺ CD127⁺), CMPs (common myeloid progenitors, Lin⁻ Sca-1⁻ c-Kit⁺ CD34⁺ CD127⁻ CD16/32⁻) was similar in Control (*n* = 11–15) and *Dlc1*^*EC-KO*^ (*n* = 8–12) mice. MEPs (megakaryocyte-erythroid progenitors, Lin⁻ Sca-1⁻ c-Kit⁺ CD34⁻ CD127⁻ CD16/32⁻) and GMPs (granulocyte-monocyte progenitors, Lin⁻ Sca-1⁻ c-Kit⁺ CD34⁺ CD127⁻ CD16/32⁺) were reduced in *Dlc1*^*EC-KO*^ mice but the reduction was not significant compared to the control mice; MEPs and GMPs: control *n* = 11, *Dlc1*^*EC-KO*^ *n* = 8 mice. ***P < 0.001; ns, not significant, by unpaired two-tailed Student’s t-test. Quantifications were obtained by flow cytometric analysis during sample acquisition. Entire bone marrow suspensions were collected and fully acquired by the flow cytometer to ensure absolute cell counts. Each dot represents data from an individual mouse. See Supplementary Fig. S3C for gating strategy. **B** Mature bone marrow hematopoietic cell populations analyzed by flow cytometry. B and T cell numbers were similar in control and *Dlc1*^*EC-KO*^ groups. The number of granulocytes and monocytes was reduced in *Dlc1*^*EC-KO*^ mice (*n* = 24) compared to control (*n* = 37). *P < 0.05, unpaired two-tailed Student’s *t*-test. Quantifications were obtained by flow cytometric analysis during sample acquisition. Entire bone marrow suspensions were collected and fully acquired by the flow cytometer to ensure absolute cell counts. Each dot represents data from an individual mouse. See Supplementary Fig. S3B for gating strategy. **C** *Dlc1* mRNA expression in BMEC transduced with lentivirus expressing a control shRNA (shCtrl) or *Dlc1*-targeting shRNAs (#1077 or #5295), confirming efficient knockdown measured by qPCR. The results reflect the means of triplicate measurements. **D** Schematic of the co-culture experiment. FACS-enriched LSK cells from tdTomato mice were co-cultured with BMEC monolayers transduced with shCtrl or sh*Dlc1* lentivirus. Each week, half of the culture medium and floating cells were harvested for analysis, and the remaining cells/media was replenished with fresh medium. At the endpoint (day 28), all floating and adherent cells were collected (after trypsinization) and pooled for flow cytometry. For quantification, all tdTomato⁻ cells were considered BMECs. Time-course quantification of tdTomato⁺ cell subsets over the 28-day co-culture; (**E**) total number of tdTomato⁺ cells; (**F**) tdTomato⁺ CD11b^+^ myeloid cells; (**G**) tdTomato⁺ CD3^+^CD19^+^ lymphoid cells; (**H**) tdTomato⁺ Lin⁻Sca1⁺c-Kit⁺ LSK cells. To account for the use of ½ of the culture medium containing floating cells on days 7, 14, and 21, the cell number on day 28 (the entire culture was used) was divided by a factor of 2. Data represent mean ± SD; *n* = 3 experiments. Quantifications were obtained by flow cytometric analysis during sample acquisition using flow cytometry counting beads. Each dot represents data from an individual experiment. **I** Number of BMEC (tdTomato⁻) per well (*n* = 9) at day 28 by flow cytometry; ns: not significant by unpaired two-tailed Student’s *t*-test. Each dot represents data from an individual well from three individual experiments. **J** Measurement of myeloblast 32D mouse cells attachment to monolayers of BMEC cells transduced with shCtrl or sh*Dlc1* lentivirus (shDlc1#1077 and shDlc1#5295). BMEC monolayers were preincubated in medium only or with mouse TNFα (20 ng/ml; 3 hours). After washing the monolayers, fluorescent (Vybrant^®^CFDA SE tracer) 32D cells were incubated in triplicate for 15 minutes and 60 minutes (37 °C) onto the monolayers. After removal of non-adherent 32D cells, fluorescence was measured by a microplate reader. The results reflect the means ± SD of 3 experiments, each performed in triplicate cultures, and are expressed as % of input cells. **K** Cytokine profiling of serum from *Dlc1*^*EC-KO*^ (*n* = 4) and control (*n* = 4) female mice. Each serum (1:20 dilution) was incubated in duplicate onto antibody arrays against 62 proteins and processed per manufacturer’s instructions (Abcam; ab133995). The results, visualized as dots by HRP-conjugated Streptavidin, were quantified by ImageJ. All results reflect group means ± SD; individual mouse samples are identified by dots. *P < 0.05, **P < 0.01, ***P < 0.001; ns: not significant by unpaired two-tailed Student’s *t*-test.

BM ECs form specialized “niches” that support hematopoietic stem and progenitor cells (HSPCs) regulating their maintenance, proliferation, and differentiation [21–24]. To assess whether endothelial *Dlc1* deletion impacts hematopoiesis, we analyzed the composition of BM hematopoietic cells. Flow cytometry (gating in Supplementary Fig. S3B, C) revealed that *Dlc1*^EC-KO^ mice had significantly reduced numbers of LSK (Lin⁻Sca-1⁺c-Kit⁺) cells and MPP3 (Lin⁻Sca-1⁺c-Kit⁺CD135^-^CD150^-^CD48^+^) multipotent hematopoietic progenitors (Fig. 4A). Consistently, mature granulocytes and monocytes were significantly reduced in *Dlc1*^EC-KO^ mice (Fig. 4B). In contrast, the number of long-term (LT) and short-term (ST) hematopoietic stem cells (HSC), MPP4, common lymphoid progenitors (CLP), megakaryocyte-erythroid progenitors (MEPs), granulocyte-monocyte progenitors (GMPs), as well as B and T lymphocytes was similar in *Dlc1*^EC-KO^ and control mice (Fig. 4A, B).

These results demonstrate that endothelial *Dlc1* is required for maintenance of the BM pool of multipotent progenitors and output of mature myeloid cells, suggesting a role of *Dlc1* in the regulation of EC niche signals essential for balanced BM hematopoiesis.

### *Dlc1* knockdown in a BM-derived endothelial cell line impairs hematopoietic support and regulates cell adhesion

To evaluate whether *Dlc1*-depleted BM ECs are defective at supporting hematopoietic cells, we used an ex vivo co-culture assay. *Dlc1* was silenced in the BMEC-Akt immortalized murine BM endothelial cell line [11] using two *Dlc1*-targeting shRNAs, which individually reduced *Dlc1* mRNA levels (Fig. 4C). After purification from the BM of mTmG mice, tdTomato^+^ LSK cells were incubated for 4 weeks onto confluent monolayers of control (shCtrl) or *Dlc1*-knockdown (sh*Dlc1*#1077) BMEC (Fig. 4D).

Over the 28-day incubation period, co-cultures containing *Dlc1*-deficient ECs yielded significantly fewer tdTomato^+^ hematopoietic cells than controls (Fig. 4E). This reduction was primarily attributable to CD11b^+^ myeloid cells (Fig. 4F), whereas CD19^+^/CD3^+^ lymphocytes (Fig. 4G) were mostly unaffected. Also, the fluorescent LSK progenitors progressively decreased in both co-cultures over time (Fig. 4H). By the end of culture, the number of viable BMEC (tdTomato⁻) recovered was comparable between groups (Fig. 4I), indicating that *Dlc1* knockdown did not affect EC viability or growth under the conditions used.

In additional experiments, we evaluated whether *Dlc1*-depletion alters EC adhesive properties. Since cell adhesion to endothelium plays an important role in leukemogenesis and drug resistance in leukemia [25], we measured adhesion of mouse 32Dcl3 (32D) myeloblastic cells to *Dlc1*-deficient and control BMEC monolayers. After 15- and 60-minutes incubation, significantly more 32D cells attached to *Dlc1*-deficient compared to control BMEC monolayers, either pre-activated or not pre-activated with TNFα (20 ng/ml) (Fig. 4J).

Next, we broadly evaluated the effects of endothelial *Dlc1* deletion on cytokine, chemokine, and other factors production (Fig. 4K). Compared to control (*n* = 4), serum samples from *Dlc1*^EC-KO^ (*n* = 4) mice showed significantly increased levels of selected cytokines, including IL-12, IL-1α, and IL-2; chemokines, including CCL2, CXCL2; and other proteins, including VCAM-1, L-selectin, and P-selectin (Fig. 4K). No serum protein was significantly decreased in *Dlc1*^EC-KO^ compared to control mice, whereas 40 additional proteins were detected at similar levels in the two groups. Overall, these results show that *Dlc1*-deficient BMECs are compromised in their ability to support hematopoietic cell expansion, particularly myeloid cell differentiation, consistent with the in vivo reduction in granulocytes and monocytes in Dlc1^EC-KO^ mice. These results also show that Dlc1-deficient BMEC are more adhesive to myeloblastic 32D cells than control BMEC, compatible with a pro-leukemic supportive niche function, and secrete/shed abnormally increased levels of cytokines, chemokines, and other factors, suggestive of systemic effects.

### *Dlc1*-deficient bone marrow endothelial cells exhibit broad transcriptomic dysregulation

**Fig. 5:**
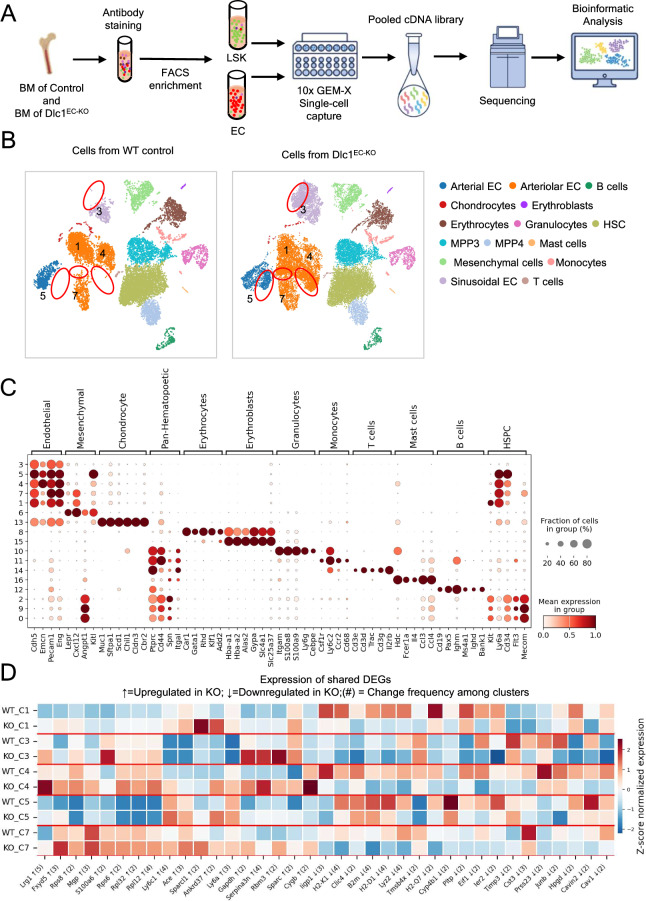
Single-cell RNA sequencing reveals altered transcriptome in Dlc1-silenced bone marrow endothelial cells. **A** Schematic overview of the experiment. Bone marrow cells from control and *Dlc1*^*EC-KO*^ mice were enriched with endothelial cells (EC) and LSK hematopoietic progenitors by cell sorting. Single-cell suspensions were processed using the 10x Genomics 3′ GEM-X platform. Libraries were pooled, sequenced on Illumina NextSeq 2000, and analyzed with CellRanger and downstream pipelines as detailed in “Materials and methods”. **B** Uniform Manifold Approximation and Projection (UMAP) plots showing unsupervised clustering and cell type annotation (indicated by coloration) of bone marrow cells from WT (top), and *Dlc1*^*EC-KO*^ (bottom) groups. **C** Cell type classification of 17 clusters into 12 major cell populations based on CellTypist annotation; dot plot of representative canonical marker genes corresponding to each annotated cell type. **D** Heatmap showing expression of shared top and bottom 20 differentially expressed genes (DEGs) across endothelial cell clusters C1, C3, C4, C5, and C7. WT identifies control WT clusters; KO identifies *Dlc1*-KO clusters. Arrows denote the direction of change (↑upregulated, ↓ downregulated in KO compared to WT), and numbers in parentheses indicate the frequency with which each gene was identified as a DEG across the five EC clusters. Z-scores represent normalized expression levels. Only genes identified as DEGs in at least two clusters (with the same directionality) are shown.

To explore how the selective *Dlc1* loss in ECs mechanistically reshapes the BM endothelial niche, we performed single-cell RNA sequencing (scRNA-seq) on enriched BM ECs and LSK progenitors from control and *Dlc1*^*EC-KO*^ mice (Fig. 5A).

Unsupervised clustering identified 16 cell populations (Fig. 5B and Supplementary Fig. S4A), including HSPCs (clusters 0, 2 and 9; Fig. 5C and Supplementary Fig. S4B); arterial, arteriolar, and sinusoidal ECs (clusters 1, 3, 4, 5 and 7; Supplementary Fig. S4C); mature hematopoietic cells of different lineages (clusters 8, 10–12, 14–16; Fig. 5C); and stromal populations (6 and 13, Fig. 5C). As expected, ECs from *Dlc1*^EC-KO^ mice showed nearly complete absence of *Dlc1* exon 5 transcripts (Supplementary Fig. S4D). Differential expression analysis revealed widespread transcriptional changes in *Dlc1*-deficient EC clusters, indicating global transcriptomic remodeling in these cells (Fig. 5D and Supplementary Fig. S4E, F).

**Fig. 6:**
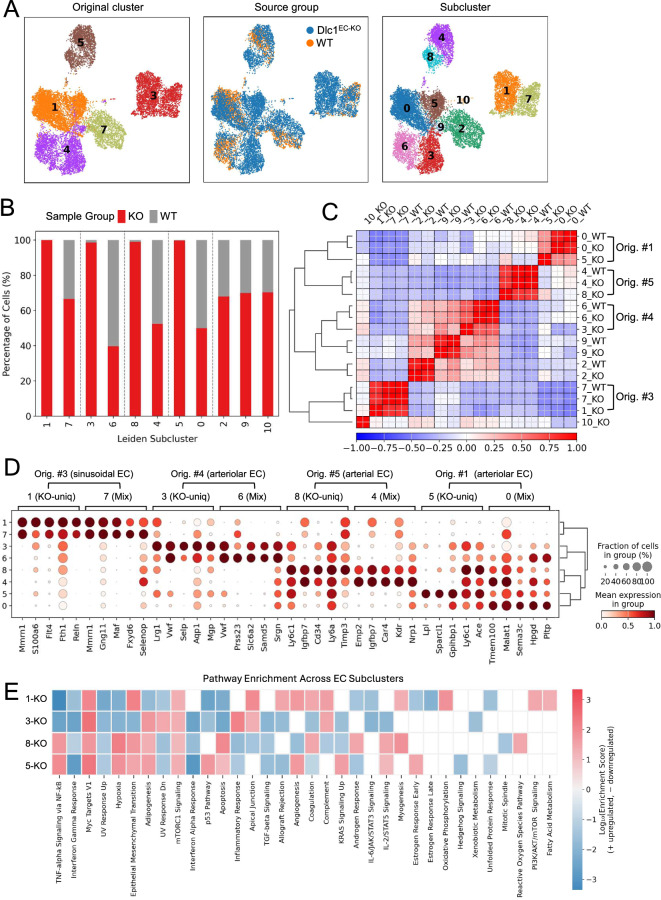
scRNA-seq identifies cell subtypes unique to Dlc1-deficient bone marrow endothelial cells. **A** UMAP plots showing re-clustering of endothelial cells. Left: original cluster assignments. Middle: source group (WT, orange; *Dlc1*^*EC-KO*^, blue). Right: subclustering of original clusters (containing WT and KO endothelial cells) reveals the KO-predominant endothelial cell subpopulations 1, 3, 8, and 5. The original cluster 3 is split into subclusters 1 (mostly KO) and 7 (mix of WT and KO); cluster 5 split into subclusters 8 (mostly KO) and 4 mix of WT and KO; cluster 1 split into subclusters 0 (mix of WT and KO) and 5 (mostly KO); and cluster 4 split into subclusters 6 (mix of WT and KO and 3 (mostly KO). **B** Bar graph showing subcluster composition. KO subclusters 1, 3, 8, and 5 are >98% KO-derived and are now defined as KO-unique. Mixed subclusters contain 30–60% WT cells. **C** Pearson correlation heatmap comparing transcriptional similarities across KO-unique and mixed subclusters composed of a mixture of WT and KO cells, which are shown individually in the heatmap. KO-unique subpopulations diverge from WT and KO components of the mixed subclusters. **D** Dot plot showing marker gene expression across endothelial cell subclusters. KO-uniq. identifies the subclusters >98% derived from KO; the mixed subclusters (Mix) contain WT- plus KO-derived endothelial cells. **E** Heatmap showing the pathways enriched across the subpopulations of KO-unique subclusters 1, 3, 8, and 5 compared to the mixed corresponding subclusters 7, 6, 4, and 0. The results from GO enrichment analysis show enrichment scores comparing KO-unique subclusters with the corresponding mixed subclusters.

Although most clusters were conserved between *Dlc1* genotypes, EC clusters 1, 3, 4 and 5 in the *Dlc1*^EC-KO^ sample appeared to contain unique subpopulations absent from the wild-type (WT) sample (Fig. 5B and Supplementary Fig. S4A). Re-clustering all ECs at higher resolution [26] uncovered several KO-specific subclusters (labeled 1, 3, 5, 8) composed almost exclusively of *Dlc1*-deficient cells (Fig. 6A–C). These KO-restricted subclusters had distinct transcriptional profiles compared to adjacent mixed subclusters (containing both WT and KO cells) (Fig. 6D and Supplementary Fig. S5A, B).

Gene set enrichment analysis comparing KO-restricted subclusters with their corresponding WT-enriched subclusters showed upregulation or downregulation of pathways linked to known DLC1 functions, including NF-κB signaling [27], Myc signaling [28], interferon response [29], inflammatory response [27], angiogenesis [7, 30], coagulation [31], and cell junction regulation [32] (Fig. 6E).

Together, these findings demonstrate that *Dlc1* loss reprograms the transcriptional landscape of BM ECs and gives rise to novel endothelial cell populations with distinct molecular identities.

### Disrupted endothelial-to-hematopoietic communication underlies impaired hematopoiesis

**Fig. 7:**
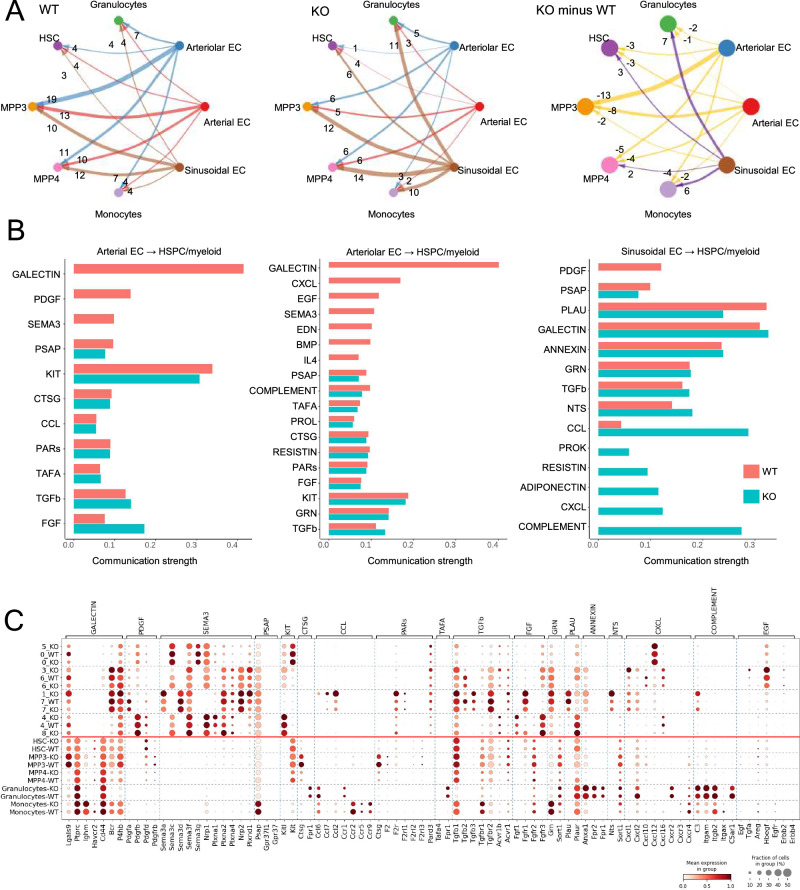
Dlc1 deficiency in bone marrow endothelial cells disrupts endothelial-to-hematopoietic cell communication. **A** Circular plots showing endothelial-to-hematopoietic cell communication patterns inferred by CellChat from transcriptomic ligand–receptor pairs. Left, WT endothelial-hematopoietic cell networks; middle, *Dlc1*^*EC-KO*^ endothelial-hematopoietic cell networks; right, network displaying gained or lost interactions in KO relative to WT (KO minus WT). In the left and middle panels, dots and line-segment coloration indicate the endothelial cell source and communication to hematopoietic cell types; line-segment width represents the number of detected signaling interactions. In the right panel, purple line-segments denote an increase, and yellow lines denote a decrease of interactions in KO relative to WT; lines width reflects the magnitude of change. **B** Bar graph summarizing the communication strength of signaling pathways linking endothelial and hematopoietic cells, as calculated by CellChat. The coral bars represent the communication strength between WT endothelial cells and WT hematopoietic cells, and the teal bars represent the communication strength between KO endothelial cells and KO hematopoietic cells; absence of communication is reflected by the absence of a bar. **C** Dot plot showing expression of representative ligands and receptors from key CellChat-identified signaling communication across endothelial cell subclusters and hematopoietic cell populations, presented separately for WT and KO groups. Dot size indicates the proportion of cells in the indicated subcluster expressing the gene; color intensity reflects mean gene expression level.

We hypothesized that the transcriptional remodeling of ECs in *Dlc1*^EC-KO^ mice impairs their ability to support hematopoietic cells [23, 25, 33, 34]. To investigate this possibility, we used CellChat to infer ligand-receptor interactions [35] and compared signaling from ECs to HSPCs in the WT (WT EC → WT HSPCs) versus the KO (KO EC → KO HSPCs) BM (Fig. 7A).

*Dlc1*-deficient arterial and arteriolar ECs exhibited a markedly reduced repertoire of interactions with all major hematopoietic cell populations (HSCs, MPPs, granulocytes, monocytes) relative to WT (Fig. 7A, right). Key niche-supportive signals were downregulated, including Galectin-9 (*Lgals9*) [36], PDGFB [37, 38], CXCL12 [39], EGF [40], KIT ligand (Kitl/SCF) [10, 41], and PSAP [42] (Fig. 7B, C; Supp. Fig. S6A, B). Semaphorin 3 C (Sema3C) [43, 44], not previously identified as a niche factor, was also reduced.

In contrast, sinusoidal ECs from *Dlc1*^EC-KO^ mice showed selective increase in interactions involving complement C3 and adiponectin, while showing reduced interactions involving PDGF (Fig. 7B, C; Supp. Fig. S6A, B). These shifts suggest that loss of endothelial *Dlc1* altered the signaling landscape in ECs, depriving HSPCs of key niche factors, including PDGFB, CXCL12 and SCF, while altering other factors.

Consistent with being recipients of reduced support, HSCs and MPP3 from *Dlc1*^EC-KO^ mice displayed reduced expression of ribosomal and mitochondrial genes, indicating diminished biosynthetic and metabolic capacity [45–50] (Supplementary Fig. S7A, B). Additionally, *Dlc1*-KO granulocytes displayed reduced expression of *Gsr* (glutathione reductase), essential for oxidative burst in myeloid cells [51].

Collectively, these findings demonstrate that loss of endothelial Dlc1 disrupts key supportive pathways for hematopoietic cells, providing insight into the mechanisms driving defective hematopoiesis in the BM of *Dlc1*^EC-KO^ mice.

## Discussion

This study establishes *Dlc1* as a critical regulator of embryonic and postnatal EC function, showing that the endothelial-specific *Dlc1* deletion recapitulates the embryonic lethality of the global *Dlc1* knockout [9] and disrupts the adult BM vasculature associated with a reduction of multipotent hematopoietic progenitors and impaired output of mature myeloid cells. These studies reveal previously unknown, essential roles of endothelial *Dlc1* in mouse development and postnatal hematopoiesis.

Transcriptional profiling shows that *Dlc1*-deficient ECs undergo widespread reprogramming marked by signatures of aberrant NF-κB [6], Myc [28], cytoskeletal [30], inflammatory [27], and junctional signaling [8] likely contributing to produce aberrant EC populations with distinct transcriptional identities and compromising production of niche factors essential for hematopoietic cell support. Consistent with this possibility, BM *Dlc1*^EC-KO^ ECs display aberrant expression of CXCL12, SCF, and other factors that were previously identified as BM niche factors [10, 39, 41]. Our analysis predicts that endothelial Sema3C is a previously unreported BM niche factor; this prediction should be further investigated. Interestingly, Semaphorins, which were originally identified as neuronal cell guidance molecules [52], are also known to control cardiovascular development [53] and to inhibit EC functions by competing with VEGF/neuropilin receptor binding [54].

Combined vascular and hematopoietic cell defects characterize clinical syndromes of idiopathic bone marrow failure. It is possible that defective hematopoiesis in *Dlc1*^EC-KO^ mice mimics early stages of Myelodysplastic Syndrome (MDS), a diverse group of myeloid malignancies characterized by clonal expansions of HSC and bone marrow failure [55]. Both mouse and human studies indicate that the endothelial cell compartment contributes to the pathology of MDS, and mouse models show that the BM vasculature exhibits structural damage and evidence of cell death in AML [25]. Additionally, *Dlc1* deficiency at diagnosis predicts poor prognosis in AML [56] and *Dlc1* methylation status pre- and post-azacytidine treatment predicts responsiveness to treatment in MDS patients [57]. We found that 32Dcl3 myeloblasts adhere more effectively to *Dlc1*-silenced endothelial cells than to control cells, a result consistent with endothelial *Dlc1* deficiency promoting a supportive leukemic niche.

In summary, endothelial *Dlc1* integrates structural and angiocrine programs that collectively maintain the vascular architecture and hematopoietic homeostasis in the BM. Loss of endothelial *Dlc1* appears to initiate a series of events where endothelial cells acquire abnormal phenotypes, alter their signaling, subvert output of niche and other factors, culminating in the selective loss of myeloid-biased hematopoietic progenitors. These findings define *Dlc1* as a key organizer of the BM vascular niche. Since *Dlc1* is often epigenetically silenced in cancer cells, and this deficiency is drug-reversible [58], further exploration of *Dlc1* deficiency in niche-driven BM failures is warranted.

## Supplementary information

Supplementary Information

## Data Availability

For original data, please contact G. Tosato at tosatog@mail.nih.gov. Raw data of single-cell RNA seq are available at Sequence Read Archive (SRA, PRJNA1355203).

## References

[1] Yuan BZ, Miller MJ, Keck CL, Zimonjic DB, Thorgeirsson SS, Popescu NC. Cloning, characterization, and chromosomal localization of a gene frequently deleted in human liver cancer (DLC-1) homologous to rat RhoGAP. Cancer Res. 1998;58:2196–9. 9605766

[2] Xue W, Krasnitz A, Lucito R, Sordella R, Vanaelst L, Cordon-Cardo C, et al. DLC1 is a chromosome 8p tumor suppressor whose loss promotes hepatocellular carcinoma. Genes Dev. 2008;22:1439–44. 18519636 10.1101/gad.1672608 PMC2418580

[3] Yang XY, Guan M, Vigil D, Der CJ, Lowy DR, Popescu NC. p120Ras-GAP binds the DLC1 Rho-GAP tumor suppressor protein and inhibits its RhoA GTPase and growth-suppressing activities. Oncogene. 2009;28:1401–9. 19151751 10.1038/onc.2008.498 PMC2715999

[4] Durkin ME, Yuan BZ, Zhou X, Zimonjic DB, Lowy DR, Thorgeirsson SS, et al. DLC-1:a Rho GTPase-activating protein and tumour suppressor. J Cell Mol Med. 2007;11:1185–207. 17979893 10.1111/j.1582-4934.2007.00098.x PMC4401278

[5] Shih YP, Yuan SY, Lo SH. Down-regulation of DLC1 in endothelial cells compromises the angiogenesis process. Cancer Lett. 2017;398:46–51. 28408355 10.1016/j.canlet.2017.04.004 PMC5460982

[6] Sanchez-Martin D, Otsuka A, Kabashima K, Ha T, Wang D, Qian X, et al. Effects of DLC1 deficiency on endothelial cell contact growth inhibition and angiosarcoma progression. J Natl Cancer Inst. 2018;110:390–9. 29202196 10.1093/jnci/djx219 PMC6059196

[7] Ritchey L, Ha T, Otsuka A, Kabashima K, Wang D, Wang Y, et al. DLC1 deficiency and YAP signaling drive endothelial cell contact inhibition of growth and tumorigenesis. Oncogene. 2019;38:7046–59. 31409902 10.1038/s41388-019-0944-x PMC8276116

[8] van der Stoel M, Schimmel L, Nawaz K, van Stalborch A-M, de Haan A, Klaus-Bergmann A, et al. DLC1 is a direct target of activated YAP/TAZ that drives collective migration and sprouting angiogenesis. J Cell Sci. 2020;133:jcs239947. 31964713 10.1242/jcs.239947

[9] Durkin ME, Avner MR, Huh CG, Yuan BZ, Thorgeirsson SS, Popescu NC. DLC-1, a Rho GTPase-activating protein with tumor suppressor function, is essential for embryonic development. FEBS Lett. 2005;579:1191–6. 15710412 10.1016/j.febslet.2004.12.090

[10] Xu C, Gao X, Wei Q, Nakahara F, Zimmerman SE, Mar J, et al. Stem cell factor is selectively secreted by arterial endothelial cells in bone marrow. Nat Commun. 2018;9:2449. 29934585 10.1038/s41467-018-04726-3 PMC6015052

[11] Poulos MG, Crowley MJP, Gutkin MC, Ramalingam P, Schachterle W, Thomas JL, et al. Vascular platform to define hematopoietic stem cell factors and enhance regenerative hematopoiesis. Stem Cell Rep. 2015;5:881–94. 10.1016/j.stemcr.2015.08.018 PMC4649106 26441307

[12] Butler JM, Nolan DJ, Vertes EL, Varnum-Finney B, Kobayashi H, Hooper AT, et al. Endothelial cells are essential for the self-renewal and repopulation of Notch-dependent hematopoietic stem cells. Cell Stem Cell. 2010;6:251–64. 20207228 10.1016/j.stem.2010.02.001 PMC2866527

[13] Dominguez Conde C, Xu C, Jarvis LB, Rainbow DB, Wells SB, Gomes T, et al. Cross-tissue immune cell analysis reveals tissue-specific features in humans. Science. 2022;376:eabl5197. 35549406 10.1126/science.abl5197 PMC7612735

[14] Ko FC, Ping Yam JW. Regulation of deleted in liver cancer 1 tumor suppressor by protein-protein interactions and phosphorylation. Int J Cancer. 2014;135:264–9. 24114040 10.1002/ijc.28505

[15] Perantoni AO, Timofeeva O, Naillat F, Richman C, Pajni-Underwood S, Wilson C, et al. Inactivation of FGF8 in early mesoderm reveals an essential role in kidney development. Development. 2005;132:3859–71. 16049111 10.1242/dev.01945

[16] Alva JA, Zovein AC, Monvoisin A, Murphy T, Salazar A, Harvey NL, et al. VE-Cadherin-Cre-recombinase transgenic mouse: a tool for lineage analysis and gene deletion in endothelial cells. Dev Dyn. 2006;235:759–67. 16450386 10.1002/dvdy.20643

[17] Gory S, Vernet M, Laurent M, Dejana E, Dalmon J, Huber P. The vascular endothelial-cadherin promoter directs endothelial-specific expression in transgenic mice. Blood. 1999;93:184–92. 9864160

[18] Palis J, McGrath KE, Kingsley PD. Initiation of hematopoiesis and vasculogenesis in murine yolk sac explants. Blood. 1995;86:156–63. 7795222

[19] Pitulescu ME, Schmidt I, Benedito R, Adams RH. Inducible gene targeting in the neonatal vasculature and analysis of retinal angiogenesis in mice. Nat Protoc. 2010;5:1518–34. 20725067 10.1038/nprot.2010.113

[20] Sorensen I, Adams RH, Gossler A. DLL1-mediated Notch activation regulates endothelial identity in mouse fetal arteries. Blood. 2009;113:5680–8. 19144989 10.1182/blood-2008-08-174508

[21] Calvi LM, Adams GB, Weibrecht KW, Weber JM, Olson DP, Knight MC, et al. Osteoblastic cells regulate the haematopoietic stem cell niche. Nature. 2003;425:841–6. 14574413 10.1038/nature02040

[22] Kiel MJ, Morrison SJ. Maintaining hematopoietic stem cells in the vascular niche. Immunity. 2006;25:862–4. 17174928 10.1016/j.immuni.2006.11.005

[23] Nombela-Arrieta C, Pivarnik G, Winkel B, Canty KJ, Harley B, Mahoney JE, et al. Quantitative imaging of haematopoietic stem and progenitor cell localization and hypoxic status in the bone marrow microenvironment. Nat Cell Biol. 2013;15:533–43. 23624405 10.1038/ncb2730 PMC4156024

[24] Baccin C, Al-Sabah J, Velten L, Helbling PM, Grunschlager F, Hernandez-Malmierca P, et al. Combined single-cell and spatial transcriptomics reveal the molecular, cellular and spatial bone marrow niche organization. Nat Cell Biol. 2020;22:38–48. 31871321 10.1038/s41556-019-0439-6 PMC7610809

[25] Boueya IL, Sandhow L, Albuquerque JRP, Znaidi R, Passaro D. Endothelial heterogeneity in bone marrow: insights across development, adult life and leukemia. Leukemia. 2025;39:8–24. 39528790 10.1038/s41375-024-02453-x PMC11717709

[26] Traag VA, Waltman L, van Eck NJ. From Louvain to Leiden: guaranteeing well-connected communities. Sci Rep. 2019;9:5233. 30914743 10.1038/s41598-019-41695-z PMC6435756

[27] Tripathi V, Popescu NC, Zimonjic DB. DLC1 suppresses NF-kappaB activity in prostate cancer cells due to its stabilizing effect on adherens junctions. Springerplus. 2014;3:27. 24683532 10.1186/2193-1801-3-27 PMC3967735

[28] Wei W, Jiang X, Shi W, Zhou J, Xu R. Dlc1 deletion activates the canonical Wnt pathway at an early stage and affects subsequent cardiac differentiation in mouse embryonic stem cells. Genes Dis. 2025:101844. 10.1016/j.gendis.2025.101844 PMC13474022 42602276

[29] Na T, Zhang K, Yuan BZ. The DLC-1 tumor suppressor is involved in regulating immunomodulation of human mesenchymal stromal /stem cells through interacting with the Notch1 protein. BMC Cancer. 2020;20:1064. 33148199 10.1186/s12885-020-07542-5 PMC7640439

[30] Shih YP, Liao YC, Lin Y, Lo SH. DLC1 negatively regulates angiogenesis in a paracrine fashion. Cancer Res. 2010;70:8270–5. 20861185 10.1158/0008-5472.CAN-10-1174 PMC2970702

[31] Shih YP, Takada Y, Lo SH. Silencing of DLC1 upregulates PAI-1 expression and reduces migration in normal prostate cells. Mol Cancer Res. 2012;10:34–9. 22064653 10.1158/1541-7786.MCR-11-0450 PMC3262057

[32] Tripathi V, Popescu NC, Zimonjic DB. DLC1 interaction with alpha-catenin stabilizes adherens junctions and enhances DLC1 antioncogenic activity. Mol Cell Biol. 2012;32:2145–59. 22473989 10.1128/MCB.06580-11 PMC3372231

[33] Kiel MJ, Yilmaz OH, Iwashita T, Yilmaz OH, Terhorst C, Morrison SJ. SLAM family receptors distinguish hematopoietic stem and progenitor cells and reveal endothelial niches for stem cells. Cell. 2005;121:1109–21. 15989959 10.1016/j.cell.2005.05.026

[34] Kusumbe AP, Ramasamy SK, Itkin T, Mäe MA, Langen UH, Betsholtz C, et al. Age-dependent modulation of vascular niches for haematopoietic stem cells. Nature. 2016;532:380–4. 27074508 10.1038/nature17638 PMC5035541

[35] Jin S, Plikus MV, Nie Q. CellChat for systematic analysis of cell-cell communication from single-cell transcriptomics. Nat Protoc. 2025;20:180–219. 39289562 10.1038/s41596-024-01045-4

[36] Jia W, Kong L, Kidoya H, Naito H, Muramatsu F, Hayashi Y, et al. Indispensable role of Galectin-3 in promoting quiescence of hematopoietic stem cells. Nat Commun. 2021;12:2118. 33837181 10.1038/s41467-021-22346-2 PMC8035175

[37] Su RJ, Li K, Zhang XB, Pan Yuen PM, Li CK, James AE, et al. Platelet-derived growth factor enhances expansion of umbilical cord blood CD34+ cells in contact with hematopoietic stroma. Stem Cells Dev. 2005;14:223–30. 15910249 10.1089/scd.2005.14.223

[38] Ramasz B, Kruger A, Reinhardt J, Sinha A, Gerlach M, Gerbaulet A, et al. Hematopoietic stem cell response to acute thrombocytopenia requires signaling through distinct receptor tyrosine kinases. Blood. 2019;134:1046–58. 31434705 10.1182/blood.2019000721

[39] Nakatani T, Sugiyama T, Omatsu Y, Watanabe H, Kondoh G, Nagasawa T. Ebf3(+) niche-derived CXCL12 is required for the localization and maintenance of hematopoietic stem cells. Nat Commun. 2023;14:6402. 37880234 10.1038/s41467-023-42047-2 PMC10600098

[40] Fang T, Zhang Y, Chang VY, Roos M, Termini CM, Signaevskaia L, et al. Epidermal growth factor receptor-dependent DNA repair promotes murine and human hematopoietic regeneration. Blood. 2020;136:441–54. 32369572 10.1182/blood.2020005895 PMC7378456

[41] Matsuoka S, Facchini R, Luis TC, Carrelha J, Woll PS, Mizukami T, et al. Loss of endothelial membrane KIT ligand affects systemic KIT ligand levels but not bone marrow hematopoietic stem cells. Blood. 2023;142:1622–32. 37562000 10.1182/blood.2022019018 PMC10733828

[42] Breiden B, Sandhoff K. Lysosomal Glycosphingolipid Storage Diseases. Annu Rev Biochem. 2019;88:461–85. 31220974 10.1146/annurev-biochem-013118-111518

[43] Pereira CF, Chang B, Gomes A, Bernitz J, Papatsenko D, Niu X, et al. Hematopoietic Reprogramming In Vitro Informs In Vivo Identification of Hemogenic Precursors to Definitive Hematopoietic Stem Cells. Dev Cell. 2016;36:525–39. 26954547 10.1016/j.devcel.2016.02.011 PMC4785845

[44] Alto LT, Terman JR. Semaphorins and their signaling mechanisms. Methods Mol Biol. 2017;1493:1–25. 10.1007/978-1-4939-6448-2_1 PMC5538787 27787839

[45] Signer RA, Magee JA, Salic A, Morrison SJ. Haematopoietic stem cells require a highly regulated protein synthesis rate. Nature. 2014;509:49–54. 24670665 10.1038/nature13035 PMC4015626

[46] Forester CM, Ruggero D. Releasing the brake on protein synthesis in hematopoietic stem cells. Cell Stem Cell. 2021;28:1183–5. 34214436 10.1016/j.stem.2021.06.003

[47] Ansó E, Weinberg SE, Diebold LP, Thompson BJ, Malinge S, Schumacker PT, et al. The mitochondrial respiratory chain is essential for haematopoietic stem cell function. Nat Cell Biol. 2017;19:614–25. 28504706 10.1038/ncb3529 PMC5474760

[48] Morganti C, Ito K. Mitochondrial Contributions to Hematopoietic Stem Cell Aging. Int J Mol Sci. 2021;22:11117. 34681777 10.3390/ijms222011117 PMC8537916

[49] Rimmelé P, Liang R, Bigarella CL, Kocabas F, Xie J, Serasinghe MN, et al. Mitochondrial metabolism in hematopoietic stem cells requires functional FOXO 3. EMBO Rep. 2015;16:1164–76. 26209246 10.15252/embr.201439704 PMC4576984

[50] Filippi MD, Ghaffari S. Mitochondria in the maintenance of hematopoietic stem cells: new perspectives and opportunities. Blood. 2019;133:1943–52. 30808633 10.1182/blood-2018-10-808873 PMC6497515

[51] Liu Y, Yan J, Meng X, Wancket L, Lintner K, Nelin L, et al. Glutathione reductase facilitates host defense by sustaining phagocytic oxidative burst and promoting the development of neutrophil extracellular traps (172.3). J Immunol. 2012;188:172.3–3. 10.4049/jimmunol.1102683 PMC3480216 22279102

[52] Tamagnone L, Artigiani S, Chen H, He Z, Ming G-l, Song H-j, et al. Plexins are a large family of receptors for transmembrane, secreted, and GPI-anchored semaphorins in vertebrates. Cell. 1999;99:71–80. 10520995 10.1016/s0092-8674(00)80063-x

[53] Gitler AD, Lu MM, Epstein JA. PlexinD1 and semaphorin signaling are required in endothelial cells for cardiovascular development. Dev Cell. 2004;7:107–16. 15239958 10.1016/j.devcel.2004.06.002

[54] Appleton BA, Wu P, Maloney J, Yin J, Liang W-C, Stawicki S, et al. Structural studies of neuropilin/antibody complexes provide insights into semaphorin and VEGF binding. EMBO J. 2007;26:4902. 17989695 10.1038/sj.emboj.7601906 PMC2099469

[55] Cazzola M. Myelodysplastic syndromes. N Engl J Med. 2020;383:1358–74. 32997910 10.1056/NEJMra1904794

[56] Li X, Qi J, Song X, Xu X, Pan T, Wang H, et al. DLC1 deficiency at diagnosis predicts poor prognosis in acute myeloid leukemia. Exp Hematol Oncol. 2022;11:74. 36258263 10.1186/s40164-022-00335-5 PMC9580124

[57] Martín I, Navarro B, Serrano A, Villamón E, Calabuig M, Solano C, et al. Impact of clinical features, cytogenetics, genetic mutations, and methylation dynamics of CDKN2B and DLC-1 promoters on treatment response to azacitidine. Ann Hematol. 2020;99:527–37. 31989250 10.1007/s00277-020-03932-8

[58] Yuan BZ, Durkin ME, Popescu NC. Promoter hypermethylation of DLC-1, a candidate tumor suppressor gene, in several common human cancers. Cancer Genet Cytogenet. 2003;140:113–7. 12645648 10.1016/s0165-4608(02)00674-x

