# Supplementary material for: Role of endothelial Dlc1 in embryonic vascular development and adult bone marrow hematopoiesis
Source: Leukemia. 2026 Jun 25;40(8):1708–20. doi: 10.1038/s41375-026-03013-1 (PMC13421314; doi:10.1038/s41375-026-03013-1)
Supplement: Supplementary file 1 — Supplementary Information [file 41375_2026_3013_MOESM1_ESM.pdf]

Supplementary Information

Role of endothelial Dlc1 in embryonic vascular development and adult bone marrow hematopoiesis

Jing-Xin Feng, Marian E. Durkin, Mei-Ting Yang, Lili Li, Ross Lake, Caiyi Li, Ferenc Livak, Lino Tessarollo, Michael Kruhlak, Douglas R. Lowy, Giovanna Tosato

Supplemental Data Contents:

1. Supplementary Methods
2. Supplementary Figures and Legends

Supplementary Methods

Chemicals and general reagents

DAPI (4′,6-Diamidino-2-Phenylindole, Dilactate; BioLegend, #422801); Tetrahydrofuran (Millipore-Sigma, #186562); Nycodenz (Accurate Chemical & Scientific Corp, #AN1002424); Urea (Millipore-Sigma, #U5378); Sodium azide (Millipore-Sigma, #S2002); Chromium GEM-X Single Cell 3′ Kit v4 (10x Genomics, #1000686); Chromium GEM-X Single Cell 3′ Chip Kit v4 (10x Genomics, #1000690); SPRIselect DNA Size Selection Reagent (Beckman Coulter, #B23317); Low-EDTA TE Buffer (Thermo Scientific, #J75793-AP); Phusion Hot Start II High-Fidelity PCR Master Mix (Thermo Scientific, #F566L); 2× PCR PreMix (Syd Labs, #MB067-EQ2B-XL); NextSeq 2000 P3 Reagents (100 cycles; Illumina, #20040559); Tamoxifen (Sigma-Aldrich, #T5648).

Antibodies

Brilliant Violet 605 anti-mouse CD31 (BioLegend, #102427); PerCP/Cyanine5.5 anti-mouse CD45 (BioLegend, #103131); BUV737 rat anti-mouse CD144 (BD Biosciences, #741792); CD34 (EP373Y) monoclonal antibody (Abcam, #ab81289); PE-Cy7 rat anti-mouse Ly-6G (BD Biosciences, #560601); BUV737 rat anti-mouse CD19 (BD Biosciences, #100234); Alexa Fluor 700 anti-mouse/human CD11b (BioLegend, #101222); APC mouse lineage antibody cocktail (BD Biosciences, #558074); BV711 rat anti-mouse CD117 (BD Biosciences, #563160); PerCP/Cyanine5.5 anti-mouse Ly-6A/E (Sca-1; BioLegend, #108124); Brilliant Violet 421 anti-mouse CD135 (BioLegend, #135314); BV510 hamster anti-mouse CD48 (BD Biosciences, #563536); APC/Fire 750 anti-mouse CD150 (SLAM; BioLegend, #115940); BV605 rat anti-mouse CD127 (BD Biosciences, #569294); Alexa Fluor 700 rat anti-mouse CD34 (BD Biosciences, #560518); BV786 rat anti-mouse CD16/CD32 (BD Biosciences, #751692).

Generation and validation of the *Dlc1*^{fl} mouse strain

The conditional *Dlc1* targeting allele was engineered to enable Cre-mediated excision of the 1427-bp exon 5 of the mouse *Dlc1* variant 2 isoform (NM_015802), which encodes isoform 2 of the mouse DLC1 protein (1092 amino acids, NP_056617).

The targeting vector was constructed from 129/Sv mouse genomic DNA, incorporating a 5.2 kb 5' homology arm (containing exon 4), a 1.9 kb inverted frt-loxP-PGK-neomycinR (neo)-bpA-frt-loxP cassette, an 8.8 kb 3' homology arm (containing exons 5-9), a single inverted loxP site in

intron 5, and a PGK-thymidine kinase cassette. The single loxP site was introduced into intron 5 via strand overlap PCR, creating a *Nhe*I site (GCTTGC > GCTAGC) for ligation of two annealed complementary loxP oligonucleotides with *Nhe*I/*Xba*I-compatible overhangs.

The linearized targeting vector was electroporated into v6.4 C57BL/6J × 129/Sv^{Jae} F1 hybrid mouse embryonic stem (ES) cells. Drug-resistant ES clones were screened for correct *Dlc1* gene targeting using long-range PCR, employing a forward primer (D46, GCA GCT TTC ATT ACA TGT GTC C) upstream of the 5' homology arm and a reverse primer (BGH, CTA TGG CTT CTG AGG CGG AAA G) in the neo cassette's 3'-UTR, yielding a 5.6 kb product in the targeted allele. The presence of the single loxP site in intron 5 was confirmed by standard PCR using primers flanking the site (D60 GCA GAC AGT GGA CCC TGG AAG; D61 GAT TTG GAA CTG CAG GAG AAT C), which distinguish the targeted (168 bp) and wild-type (121 bp) alleles.

A correctly targeted ES clone (*Dlc1*^{neo}) was injected into C57BL/6 blastocysts to generate chimeric mice, which were then bred to wild-type C57BL/6 mice for germline transmission. Genotyping was performed using primers for the loxP site in intron 5 (D60/D61) and the intron 4/neo cassette junction (D49, GAC GTA CTC CTG ATG GTT GAG AG, and BGH).

To remove the frt-flanked neo cassette, *Dlc1*^{neo} mice were mated with β-actin promoter-FLPe recombinase transgenic mice (1) (JAX, # 005703), producing the "floxed" allele (*Dlc1*^{fl}). Excision of the neo cassette was verified by PCR with primers (D49 and D2, CGA GTA CAC GGT ATC ACA CA) flanking the genomic DNA/neo cassette junctions in intron 4, resulting in 232-bp (*Dlc1*^{fl}) and 132-bp (*Dlc1*^{wt}) products.

To functionally validate that deletion of exon 5 results in a null allele, *Dlc1*^{neo} mice were bred to T-Cre transgenic mice (MGI # 3605847) to generate the *Dlc1*$^{\Delta 5}$ allele. The offspring were genotyped using PCR primers in intron 4 and intron 5 (D49/D61) that amplify a 116-bp product after excision of the neo cassette and exon 5. Mice lacking exon 5 were back-crossed to C57BL/6 mice to ensure germline transmission of the *Dlc1*$^{\Delta 5}$ allele. RT-PCR analysis of RNA from the tissues of *Dlc1*$^{\Delta 5/wt}$ and *Dlc1*$^{wt/wt}$ mice was performed with primers in exon 3 (GCT GGT CAA GAG AGA ACA TG) and exon 7 (GTC CTT GTA GTC TGG AAC CTT G), which yielded a 347-bp product from the *Dlc1*$^{\Delta 5}$ transcript, in contrast to the 1774-bp product from the transcript of the wild type *Dlc1* allele. Sanger sequencing of the 347-bp product confirmed splicing of exon 4 to exon 6, resulting in a reading frameshift after Arg85 of the DLC1 protein, which leads to premature translation termination.. The mutant transcript is predicted to encode a severely shortened protein with 23 aberrant amino acids at the C-terminus, indicating that exon 5 deletion produces a functionally null allele.

Animal husbandry

All animal procedures were approved by the Institutional Animal Care and Use Committee of the Center for Cancer Research (CCR, Bethesda, MD), National Cancer Institute (NCI), NIH, and conducted in accordance with the NIH Guide for the Care and Use of Laboratory Animals (National Academies Press, 2011) and approved institutional protocols.

Cdh5-CreERT2 mice (MGI:3848982) were generously provided by Dr. Ralf Adams. Cdh5-Cre(7Mlia) mice(2) (JAX #006137), Cre-dependent Ai6-ZsGreen(3) (JAX #007906), and mTmG (4) (JAX #007676) fluorescent Cre-reporter strains were obtained from The Jackson Laboratory. All lines were maintained on a C57BL/6J genetic background and housed in CCR/NCI animal facilities under specific pathogen-free conditions.

Mice were identified by ear tagging and routinely genotyped by PCR. No animals were excluded from experiments unless they were deemed ill by veterinary staff or exhibited fight-related injuries at the time of tissue harvest.

Mouse genotyping

For adult mice, tail clips (~1mm) were collected at weaning for genotyping. For embryos, yolk sac samples free of maternal blood were collected at the time of harvest. Genomic DNA was extracted using the HotSHOT method. Briefly, samples were incubated in alkaline lysis buffer (25 mM NaOH, 0.2 mM EDTA in water) at 95°C for 2 hours, followed by neutralization with an equal volume of 40 mM Tris-HCl (pH 5.0). Samples were mixed thoroughly after neutralization and centrifuged at 15,000 × g for 2 min. The supernatant was transferred to new tubes and diluted 1:1 with water. Typically, 2 µL of DNA was used per 20 µL PCR reaction.

Genotyping primers and amplicon sizes were as follows (5' – 3'): *Dlc1*-floxed, GCA GAC AGT GGA CCC TGG AAG; GAT TTG GAA CTG CAG GAG AAT C; WT band: 121 bp; floxed band: 155 bp. *Cdh5-Cre (7Mlia)*, CTA GGC CAC AGA ATT GAA AGA TCT; GTA GGT GGA AAT TCT AGC ATC ATC C; GCC TGC ATT ACC GGT CGA TGC AAC GA; GTG GCA GAT GGC GCG GCA ACA CCA TT, Internal control: 335 bp; Tg band: 710 bp (zygosity not distinguished). *Cdh5-Cre^{ERT2}*, TCC TGA TGG TGC CTA TCC TC; CCT GTT TTG CAC GTT CAC CG; CAC CCT GTT CTT TGC CTC CT, Internal control: 422 bp; Tg band: 492 bp (zygosity not distinguished). *mTmG*, CTT TAA GCC TGC CCA GAA GA; TAG AGC TTG CGG AAC CCT TC; AGG GAG CTG CAG TGG AGT AG, WT band: 220 bp; Tg band: 135 bp. *ZsGreen*: AAG GGA GCT GCA GTG GAG TA; CCG AAA ATC TGT GGG AAG TC; GGC ATT AAA GCA GCG TAT CC; AAC CAG AAG TGG CAC CTG AC, WT band: 300 bp; Tg band: 210 bp.

Tamoxifen administration

Tamoxifen (Sigma-Aldrich, #T5648) was dissolved in peanut oil (Sigma-Aldrich, #P2144) at a concentration of 10 mg/mL and administered via oral gavage using 22 G feeding needles at a dose of 100 $mg \cdot kg^{-1}$ to adult mice. Tamoxifen together with progesterone (50 mg/kg^{-1}) was administered by oral gavage (100 $mg \cdot kg^{-1}$ per day × 3 days) to pregnant females at E8.5–E10.5. Unless otherwise specified, three consecutive daily doses were given. Both male and female mice aged 4–8 weeks were used unless stated otherwise. No randomization or blinding was applied during group allocation. Mice were typically sacrificed between 9 AM and 11 AM local time.

Timed pregnancy and tamoxifen administration to pregnant females

To obtain embryos from timed pregnancies, one Cdh5-Cre^{+}/*Dlc1*$^{fl/+}$ male and one Cdh5-Cre^{+}/*Dlc1*$^{fl/+}$ female were paired from 4:30 PM to 8:00 AM the next day, which was designated as embryonic day (E) 0.5. The body weight (BW) of females was recorded, and the presence of a copulation plug was checked the next morning. BW was monitored over the following week. Pregnancy was confirmed by the presence of a copulation plug combined with a BW increase of more than 1 gram. Confirmed pregnant females were administered tamoxifen at 100 $mg \cdot kg^{-1}$ (20 $mg \cdot mL^{-1}$ in peanut oil) and 50 $mg \cdot kg^{-1}$ progesterone (20$mg \cdot mL^{-1}$ in peanut oil), starting at E8.5 or E10.5, once daily for three consecutive days. Embryos were harvested at E11.5 or E13.5.

Embryo harvest

Pregnant dams were euthanized at the indicated time points by cervical dislocation. Embryos were dissected individually from the decidua in ice-cold PBS. After yolk sac removal, embryos were imaged using a stereomicroscope and a mobile phone camera (iPhone XS Pro Max). Yolk sacs free of maternal blood were collected for genotyping (see Mouse genotyping for details).

Blood collection and CBC test

For terminal collection, blood was obtained from the mouse abdominal aorta with BD Vacutainer™ EDTA tubes (BD #367856) and Vaculet™ blood collection needles (23G, EXELINT #26766). For flow cytometry analysis, ACK buffer (Lonza, #BP10-548E) was added to the blood to lyse red blood cells before Fc receptor blocking and antibody staining. For non-terminal blood collection, ~20-50µL blood was collected by submandibular blood sampling, using a 3mm animal lancet (BRAINTREE SCI., GR-3MM) and a 250µL BD Microtainer® K2EDTA tube (BD #365974). Kwik Stop® Styptic Powder was applied to stop the bleeding

(Miracle Corp. #423615). For long-term blood collection, tail vail sampling was used. White blood cell (WBC) counts were determined using acridine orange/propidium iodide (AO/PI, Logos Biosystems, #F23001) staining and quantified with a LUNA-FL™ fluorescence cell counter (Logos Biosystems). CBC tests were performed at the Department of Laboratory Medicine, NIH Clinical Center. Serum samples were derived from coagulated blood by standard techniques.

Bone marrow harvest for flow cytometry analysis

For flow cytometry analysis, bone marrow was harvested using one of the two methods described below. For hematopoietic cell isolation, bone marrow was harvested by flushing femurs and tibiae with ice-cold Sort Buffer (1× PBS [Gibco, #10010-031] supplemented with 5 mM EDTA, 25 mM HEPES, and 2% FBS [Sigma-Aldrich, #F2442]). Red blood cells were then lysed using ACK lysing buffer (Lonza, #10-548E) according to the manufacturer's instructions. Cells were then washed with Sort Buffer and filtered through a 40 μm cell strainer (Greiner Bio-One, #542040, #542140). For endothelial cell preservation, bone marrow was harvested by gently crushing femurs and tibiae in Sort Buffer (1× PBS with 5 mM EDTA, 25 mM HEPES, and 2% FBS). Red blood cell lysis was performed using ACK buffer. The cell suspension was then incubated with 0.1 U/mL Collagenase (Worthington Biomedical Corp., #LS004176), 0.8 U/mL Dispase (Worthington Biomedical Corp., #LS02109), and 0.5 U/mL DNase I (Worthington Biomedical Corp., #LS006344) in 1× Hanks' Balanced Salt Solution (HBSS) with Ca^{2+} and Mg^{2+} (Gibco, #14065056) at 37°C for 30 minutes on a rotating mixer. Cells were subsequently washed with Sort Buffer and filtered through a 40 μm strainer.

Flow cytometry and cell sorting

Single-cell suspensions of bone marrow and blood were first incubated with azide-free Fc receptor blocker (Innovex, #NB335-60) in Sort Buffer (1× PBS containing 5 mM EDTA, 25 mM HEPES, and 2% FBS), following the manufacturer's instructions. After washing, cells were stained with surface marker antibodies at a concentration of 2 μg per 1×10^7 cells in Sort Buffer for 30 minutes at 4°C. After staining, cells were washed and resuspended in Sort Buffer containing either propidium iodide (PI, 0.5 μM, Millipore Sigma, #P4170), 7-AAD (1 μg/mL, Millipore Sigma, #A9400), or DAPI (0.5 μg/mL, BioLegend, #422801) to discriminate live from dead cells. For FACS sorting of live endothelial cells, $CD45^+$ cells were first depleted using MojoSort Mouse CD45 nanobeads (BioLegend, #480028) following the manufacturer's protocol, followed by antibody staining. For flow cytometry analysis, compensation beads (BD Biosciences, #552844) were used to perform multicolor compensation. Flow cytometric data were acquired using BD FACSCanto II, BD LSRFortessa, BD FACSymphony A5 (BD

Biosciences), Sony SA3800, and Sony ID7000 cell analyzers. Cell sorting was performed using BD FACSAria III, BD FACSAria Fusion, or Sony SH800S sorters. Forward and side scatter (FSC and SSC) parameters were used to exclude debris and dead cells. Viability dyes (7-AAD, PI, DAPI, or BioLegend Zombie Dye) were used to exclude dead cells. When counting was needed, counting beads (BD #335925) were used during sample acquisition. Single cells were gated based on FSC-W versus FSC-H and SSC-W versus SSC-H. Unless otherwise stated, fluorescence-minus-one (FMO) controls were used to define negative gating thresholds. For bone marrow HSPC analysis, lineage-positive cells were depleted using the Mouse Lineage Cell Depletion Kit (BioLegend, #480004) prior to staining. Data were analyzed using FlowJo (BD, v10.8.1), Sony ID7000 Software (v1.2.0.28212), or BD FACSDiva (v6.1 and v9.0).

Bone marrow cryosections

Deeply anesthetized mice were transcardially perfused with 20 mL of ice-cold 1× PBS, followed by 15 mL of ice-cold hydrogel solution (5% acrylamide/bis-acrylamide 19:1 [Sigma-Aldrich, #A2917], 2.5 mg/mL polymerization initiator VA-044 [FUJIFILM Wako, VA-044], and 4% PFA in 1× PBS) at a flow rate of 5 mL/min. Femurs and tibiae were harvested and transferred into tubes containing 5 mL of hydrogel solution, then incubated at 4°C for 4 hours. Bones were subsequently rinsed with PBS and incubated at 37°C for 2 hours to allow polymerization. Decalcification was performed by incubating bones in 40 mL of 0.5 M EDTA (pH 8.0; KD Medical, #RGF-3130) for 3 days on a rotating mixer, with fresh EDTA solution replaced daily. Bones were then dehydrated overnight in 20% sucrose and 2% polyvinylpyrrolidone in PBS. Dehydrated bones were embedded in OCT compound (SAKURA, #4583) using the Precision Cryoembedding System (IHC WORLD, #IW-P101). Cryosections (10 µm) were cut from frozen OCT-embedded bone blocks using a Leica CM3050S cryostat equipped with low-profile blades (Leica 819, #14035838925) and mounted on TruBond™ 380 adhesion slides (Electron Microscopy Sciences, #63701-W10).

Tissue clearing and whole mount imaging

Tissue clearing was performed using the EZ Clear protocol (5). Briefly, adult mice were deeply anesthetized with isoflurane inhalation, and the chest cavity was opened to expose the beating heart. The right atrium was incised, and transcardial perfusion was performed through the left ventricle using 10 mL of room-temperature 1× PBS, followed by 10 mL of cold 4% PFA. Organs were dissected and post-fixed in 4% PFA at 4°C overnight with gentle agitation. After fixation, organs were washed in 1× PBS three times (30 min each at room temperature) and stored in 1× PBS containing 0.05% sodium azide at 4°C until clearing. Adult long bones were decalcified in 0.5 M EDTA for 3 days with daily solution changes following perfusion and overnight fixation.

Embryos were harvested and fixed in 4% PFA overnight before tissue clearing. After perfusion and fixation, samples were placed in individual glass vials, wrapped in foil for light protection, and rocked on an orbital shaker at 4°C. Samples were then incubated in 20 mL of the following EZ Clear solutions sequentially: (1) Lipid removal: 50% (v/v) tetrahydrofuran (THF) with 250 ppm BHT (Millipore-Sigma, #186562) for 48 hours. (2) Wash: rinsed with distilled water four times, 1 hour each at room temperature. (3) Refractive index (RI) matching: incubated in 5 mL of EZ View mounting and imaging solution for 24 hours. EZ View consisted of 80% Nycodenz (Accurate Chemical & Scientific Corp, #AN1002424), 7 M urea, and 0.05% sodium azide, prepared in 0.02 M sodium phosphate buffer. The refractive index of EZ View was measured using a refractometer (Atago, PAL-RI3850) and ranged from 1.512 to 1.518. Cleared tissue samples were imaged using a Nikon SoRa spinning disk confocal microscope equipped with 4x plan-apochromat (N.A. 0.20) and 10x plan-apochromat (N.A. 045) objective lenses and a Hamamatsu ORCA Fusion BT sCMOS camera. Images were reconstructed and analyzed using either Imaris or ZEN image analysis software programs.

Histology and Immunohistochemistry

Mouse embryos were harvested and immediately fixed in 10% neutral buffered formalin for 72 h at room temperature. Fixed tissues were processed into paraffin using a Tissue-Tek Prisma system (Sakura Finetek, USA), sectioned at 5 µm, and mounted on glass slides. Sections were deparaffinized in xylene, rehydrated through graded ethanol (100%–75%), and rinsed in distilled water. For antigen retrieval, slides were immersed in Tris-EDTA buffer (pH 9.0) and heated to 98 °C for 20 min using a microwave, then cooled under running water for 10 min.

Endogenous peroxidase activity was blocked with 3% H_2O_2 for 10 min, followed by a 1-hour incubation in protein blocking buffer (5% rabbit serum in TBST). Sections were then incubated overnight at 4 °C with anti-CD34 (EP373Y) rabbit monoclonal antibody (1:100; Abcam, #ab81289) diluted in TBST containing 1% BSA. After three TBST washes, sections were incubated for 30 min at room temperature with an HRP-conjugated goat anti-rabbit secondary antibody (Abcam, # ab6721), developed with DAB chromogen, and counterstained with hematoxylin. Slides were scanned at 20× magnification using an Aperio AT2 digital slide scanner (Leica Biosystems, Buffalo Grove, IL). Kidney sections from adult C57BL/6J mice served as positive controls, and an isotype-matched rabbit IgG was used as a negative control.

Immunofluorescence staining and imaging

Tissue sections were rehydrated in 1× PBS for 15 minutes, permeabilized in 0.3% Triton X-100 (Sigma-Aldrich, #T9284) in PBS for 15 minutes, washed with PBS, and incubated for 2 hours in blocking solution (2% BSA, 5% donkey serum [Sigma, #D9663], and 0.3% Triton X-100 in

PBS). After three PBS washes, samples were incubated with primary antibodies (5 ng/mL) at 4°C overnight. When secondary antibodies were used, cells were washed three times with PBS before incubation with fluorophore-conjugated secondary antibodies (2 ng/mL) for 2 hours at room temperature. Afterward, samples were washed three times (10 minutes each) in 1× PBS, and counterstained with DAPI (300 nM in PBS, 10 minutes). Three additional PBS washes (5 minutes each) were performed before mounting coverslips (EPREDIA, #9990402), air-drying, and sealing with nail polish. Confocal imaging was performed using Zeiss LSM 780, Zeiss LSM 880 NLO Two Photon, or Nikon ECLIPSE Ti2-E SoRa systems, selected according to experimental requirements (e.g., resolution, speed, wavelength capabilities). For H&E slides, Zeiss Axioscan 7 systems were used for imaging of the whole slides. Image processing was performed using Zen (Zen Black v2.3, Version 14.0.12.201; Zen Blue Lite v2.5, Carl Zeiss), Bitplane Imaris (v9.7.0, Oxford Instruments), and Adobe Photoshop (v23.3.0) for adjustments to overall image brightness and contrast.

Bone marrow vessel segment counting, vessel diameter measurement and vessel coverage calculation

Quantitative analyses of bone marrow vascular architecture were performed on confocal images using a combination of open-source image processing and quantitative analysis tools, including *scikit-image* (6) (version 0.24.0), *Cellpose* (7, 8) (version 3.0), *CellProfiler* (9) (version 4.3), and *vessel_metrics* (10) (version 1.2). The source python script is available on GitHub (https://github.com/TosatoLab/BoneVesselQuantification).

Confocal images were first converted to 8-bit grayscale and subjected to illumination and background correction using a white top-hat filter (structuring element radius = 40–60 pixels) implemented in *scikit-image*. To reduce noise, a Gaussian filter (σ = 1–2 pixels) was applied before thresholding.

Vascular segmentation was performed using Cellpose with the “cyto2” pretrained model, which accurately delineated tubular endothelial structures. The model was run with flow_threshold = 0.4, cellprob_threshold = 0.0, and adaptive diameter estimation to accommodate variable vessel calibers. Resulting binary masks were visually inspected and refined in CellProfiler (IdentifyPrimaryObjects and Morph modules) to close small gaps and smooth edges.

Binary vessel masks were skeletonized in scikit-image and converted into graph representations in which *branch points* and *endpoints* were defined by 8-neighbor voxel connectivity. Vessel segments were identified as skeleton paths connecting these nodes, excluding terminal fragments <5 µm to avoid noise artifacts.

Vessel diameters were measured using two complementary approaches:

1. *Distance transform*: the local radius at each skeleton voxel was defined as the distance to the nearest background pixel (diameter = 2 × radius).
2. *Cross-sectional profiling*: using vessel_metrics, orthogonal line sampling along the skeleton centerline determined the *full width at half maximum (FWHM)* of the fluorescence intensity profile. Sigma parameters for the Meijering vesselness filter were tuned to expected vessel scales (σ_1 = 2–8 µm, σ_2 = 10–20 µm). Mean and median diameters per field were then computed.

The total number of vessel segments was determined from the skeleton graph after pruning terminal segments shorter than 10 µm. Branch points were defined as nodes with ≥3 neighbors, and segment counts were normalized to the imaged area (segments/mm^2). Graph visualizations were generated using matplotlib and vessel_metrics utilities.

Vessel coverage was defined as the fraction of the image area occupied by vessels. The binary vessel mask generated during segmentation was used to compute coverage as:
$Vessel\ coverage\ (\%) = \frac{A_{vessel}}{A_{ROI}} \times 100$, where A_{vessel} is the total number of vessel pixels and A_{ROI} is the total pixels of region of interest area. Measurements were performed on multiple non-overlapping regions per mouse to minimize sampling bias.

Cell culture

BMEC-Akt1 cells(11), a gift of Dr. J Butler, were maintained in DMEM/F-12 (Gibco, #11320-033) supplemented with 20% FBS (Sigma-Aldrich, #F2442), 10 mM HEPES (Gibco, #15630080), 100 ng/mL heparin (Millipore Sigma, #H3149), MEM Non-Essential Amino Acids (Gibco, #11140050), 50 µg/mL endothelial cell growth supplement (Corning, #356006), Penicillin-Streptomycin (Gibco, #15140-122), and Anti-Anti (Gibco, #15240-062). Cells were detached using Accutase (Gibco, #A1110501) for passaging. The mouse myeloblast 32Dcl3 cell line (12), was maintained in IMDM (Gibco, #12440-046) supplemented with 10% FBS and 10 ng/ml mouse IL-3 (R&D Systems, #403-ML/CF). HEK293T cells were cultured in complete DMEM (Gibco, #11965092) supplemented with 10% FBS and Penicillin-Streptomycin. Trypsin-EDTA (0.25%) (Gibco, #25200056) was used to detach HEK293T cells from the culture surface. All cells were cultured at 37°C in a humidified incubator with 5% CO_2.

Generation of BMEC infected with sh*Dlc1* constructs

shRNA constructs targeting *Dlc1* were purchased from Sigma (TRCN0000251077, TRCN0000251075, TRCN0000251078, TRCN0000265295, TRCN0000251076). A non-targeting control shRNA (SHC002) was also obtained from Sigma. Lentivirus was produced by

transfecting HEK293T cells (3.8×10^6 cells per 75 cm^2 flask) with *Dlc1* or control shRNA plasmids (1.64 pmol), psPAX2 (1.3 pmol), and pMD2.G (0.72 pmol) using PEI (56.7 µg) for 18 hours. The next morning, the medium containing PEI and plasmids was aspirated and replaced with 10 mL of fresh complete DMEM. Virus-containing supernatants were collected at 48-, 72-, and 96-hours post-transfection. At the final harvest, HEK293T cells were lysed in 0.5% Triton X-100 in PBS (15 minutes on ice, with pipetting up and down every 3 minutes). Cell debris was removed by centrifugation at 20,000 × g for 5 minutes. The supernatant was filtered through a 0.45 µm PES filter and then concentrated by ultracentrifugation at 48,000 × g for 2 hours at 4°C. The viral pellet was resuspended in 200 µL DMEM, aliquoted into 20 µL portions, and stored at -80°C until use. BMECs were infected with either control or *Dlc1* shRNA lentivirus and selected with puromycin (3 µg/mL; Gibco, #A1113803) for more than 4 weeks and were kept in puromycin containing medium.

Cell adhesion assay

Monolayer cultures of shcontrol and shDlc1 BMEC were established in 96-well flat-bottom culture plates by seeding $5x10^3$ or $1.5x10^4$ cells/well. After overnight incubation, culture medium was replaced with fresh medium with or without mouse TNFα (20 ng/ml; R&D System, #210-TA) for 3 hours followed by washing to remove unbound TNFα. For fluorescent labeling, 32Dcl3 cells were incubated with CFDA SE dye at 37 ºC for 15 min in the dark (Vybrant® CFSA SE Cell Tracer kit (Invitrogen, #V12883), followed by washing to remove excess dye, suspended in complete IMDM, and incubated for 30 min at 37 ºC. The fluorescent-labeled 32D cells were added ($1x10^5$) to the BMEC monolayers and then incubated for 15 or 60 min. Non adherent cells were removed by warm PBS washes. Adherent 32Dcl3 were quantified by measuring fluorescence intensity in a microplate reader (BioTek, Synergy H1, excitation 492 nm, emission 517 nm). Absorbance values were converted to cell numbers. Background fluorescence was derived from BMEC-only cultures and maximal fluorescence was derived from labeled ($1x10^5$) 32Dcl3 cells. The results are expressed as % 32Dcl3 attached of input cells.

Cytokine profiling of mouse serum samples

We used the Mouse Cytokine Antibody Array Membrane (62 targets; Abcam, ab133995) to measure cytokine levels in serum samples of female $Dlc1^{EC-KO}$ and control adult (16 week-old) mice. Serum samples diluted (1:20) in sample diluent, were spotted on the membrane, blocked with blocking buffer (30 min, room temperature), and then incubated overnight at 4 ºC. After washes, the membranes were incubated with HRP-conjugated streptavidin (2 hours at room temperature, with gentle rocking). After final washes, chemioluminescence was detected by imaging (GE, Amersham imager 680) after 2 min incubation with detection reagents at room

temperature. Several exposures ensured that signals were within the linear detection range. All membranes were processed and imaged together under the same conditions to enable comparative analyses. Signal intensities were quantified by ImageJ. Background signals were subtracted and positive control spots were used to correct inter-membrane variability.

Quantitative reverse transcription-polymerase chain reaction (qPCR)

Total RNA was extracted using the RNeasy Plus Micro Kit (Qiagen, # 74004) following the manufacturer's instructions. Between 200 ng and 500 ng of total RNA was used for reverse transcription (RT) using SuperScript III First-Strand Synthesis System (Thermo Fisher, #11752-050), according to the manufacturer's instructions. Quantitative PCR reactions were performed in technical triplicates using FastStart Universal SYBR Green Master Mix (Rox) (Roche, #04913914001) supplemented with 5 µM primers and 0.5 µL of each cDNA sample on a 384-well QuantStudio™ 5 Real-Time PCR System (Applied Biosystems). Primer sequences: *Gapdh*, AGG TCG GTG TGA ACG GAT TTG (forward), TGT AGA CCA TGT AGT TGA GGT CA (reverse); *Dlc1,* GGA CAC CAT GAT CCT AAC ACA AA (forward), AGC GCA ATA TCA ACA GGG AAC (reverse). Gene expression levels were calculated using the $2^{-\Delta\Delta Ct}$ method, with Ct values determined by QuantStudio software (v1.3). *Gapdh* expression was used as the internal control, and relative fold changes were calculated against control sample expression.

Ex vivo co-culture of bone marrow LSK cells with BMEC monolayers

LSK cells were sorted from the bone marrow of *mTmG* mice and seeded onto confluent BMEC monolayers (cultured free of puromycin starting two days before LSK seeding) at a density of 20,000 cells per well in a 6-well plate. Co-cultures were maintained in StemSpan medium (STEMCELL technologies # 09605), with 10 ng ml^{-1} mouse SCF (PeproTech, #250-03). Each week, half of the culture supernatant (containing non-adherent floating cells) was collected for analysis, and an equal volume of fresh medium was added to replenish the cultures. At week 4, all remaining cells in the well—including adherent BMEC-Akt cells—were harvested and analyzed. To ensure comparability with earlier time points, the total number of $tdTomato^{+}$ cells at week 4 was multiplied by a factor of 0.5.

Single-cell analysis

Bone marrow cells from tamoxifen-treated *Cdh5-Cre^{ERT2}/ZsGreen/Dlc1-lox* mice were enriched for $CD45^{-}CD31^{+}ZsGreen^{+}$ endothelial cells and $lineage^{-}$, $Sca\text{-}1^{+}$, $cKit^{+}$ LSK cells by FACS. Single-cell capture was performed using the 10x Genomics Chromium GEM-X Single Cell 3′

Reagent Kits (v4), following the manufacturer's protocols. Following reverse transcription (RT) in droplets, pooled cDNA was amplified for transcriptome library construction. Initial library quality control was performed using the Agilent TapeStation D5000 system. 10 μL (25%) of the 10x cDNA library was fragmented and used to generate a gene expression library, following protocols in the Single Cell 3′ Reagent Kits v4. Libraries were sequenced on an Illumina NextSeq 2000 using the P3 kit (28 + 74 bp read length). Transcript alignment was performed using 10x cellranger (v9.0) and the mouse reference genome (mm10, version 2020-A). Downstream analysis and visualizations were performed using Scanpy (https://github.com/scverse/scanpy). Batch correction was conducted using the BBKNN method (https://github.com/Teichlab/bbknn). Cell type annotation was performed using celltypist (13), using the embedded Immune_All_Low model (https://www.celltypist.org/) and verified with canonical cell type markers. For *Dlc1* exon-specific read mapping, BAM files from the 10x cellranger output were used. Endothelial cell indices were extracted to isolate EC-specific reads. Reads were aligned to the *Dlc1* genomic loci using pysam (v0.23.3). This work utilized computational resources from the NIH HPC Biowulf cluster (http://hpc.nih.gov) and the Frederick Research Computing Environment (FRCE). Analyses were conducted using Python v3.14 and R v4.5.0.

Quantification and statistical analysis

No statistical methods were used to predetermine sample size in experiments in vitro and in mice. No data or mice were excluded from inclusion in analysis. Samples or mice with the appropriate genotypes were randomly assigned to control or treatment groups. The Investigators were not blinded to group allocations of samples or mice. Unless otherwise indicated, data are presented as mean ± standard deviation (SD), and individual dots in graphs represent individual mice. Comparisons between two groups were performed using two-tailed unpaired Student's *t*-tests after verifying that the data were normally distributed. Statistical analyses were conducted using GraphPad Prism (v9.0.1). P values < 0.05 were considered statistically significant. Significance is annotated as follows: ns, not significant; *$P < 0.05$; **$P < 0.01$; *** $P < 0.001$.

References

1. Rodríguez CI, Buchholz F, Galloway J, Sequerra R, Kasper J, Ayala R, et al. High-efficiency deleter mice show that FLPe is an alternative to Cre-loxP. Nature genetics. 2000;25(2):139-40.
2. Alva JA, Zovein AC, Monvoisin A, Murphy T, Salazar A, Harvey NL, et al. VE-Cadherin-Cre-recombinase transgenic mouse: a tool for lineage analysis and gene deletion in endothelial cells. Dev Dyn. 2006;235(3):759-67.

3. Madisen L, Zwingman TA, Sunkin SM, Oh SW, Zariwala HA, Gu H, et al. A robust and high-throughput Cre reporting and characterization system for the whole mouse brain. Nat Neurosci. 2010;13(1):133-40.
4. Muzumdar MD, Tasic B, Miyamichi K, Li L, Luo L. A global double-fluorescent Cre reporter mouse. Genesis. 2007;45(9):593-605.
5. Hsu CW, Cerda J, 3rd, Kirk JM, Turner WD, Rasmussen TL, Flores Suarez CP, et al. EZ Clear for simple, rapid, and robust mouse whole organ clearing. Elife. 2022;11:e77419.
6. van der Walt S, Schonberger JL, Nunez-Iglesias J, Boulogne F, Warner JD, Yager N, et al. scikit-image: image processing in Python. PeerJ. 2014;2:e453.
7. Pachitariu M, Stringer C. Cellpose 2.0: how to train your own model. Nature Methods. 2022;19(12):1634-+.
8. Stringer C, Pachitariu M. Cellpose3: one-click image restoration for improved cellular segmentation. Nat Methods. 2025;22(3):592-9.
9. Stirling DR, Swain-Bowden MJ, Lucas AM, Carpenter AE, Cimini BA, Goodman A. CellProfiler 4: improvements in speed, utility and usability. BMC Bioinformatics. 2021;22(1):433.
10. McGarry SD, Adjekukor C, Ahuja S, Greysson-Wong J, Vien I, Rinker KD, Childs SJ. Vessel Metrics: A software tool for automated analysis of vascular structure in confocal imaging. Microvasc Res. 2024;151:104610.
11. Poulos MG, Crowley MJP, Gutkin MC, Ramalingam P, Schachterle W, Thomas JL, et al. Vascular Platform to Define Hematopoietic Stem Cell Factors and Enhance Regenerative Hematopoiesis. Stem Cell Reports. 2015;5(5):881-94.
12. de la Luz Sierra M, Sakakibara S, Gasperini P, Salvucci O, Jiang K, McCormick PJ, et al. The transcription factor Gfi1 regulates G-CSF signaling and neutrophil development through the Ras activator RasGRP1. Blood. 2010;115(19):3970-9.
13. Dominguez Conde C, Xu C, Jarvis LB, Rainbow DB, Wells SB, Gomes T, et al. Cross-tissue immune cell analysis reveals tissue-specific features in humans. Science. 2022;376(6594):eabl5197.

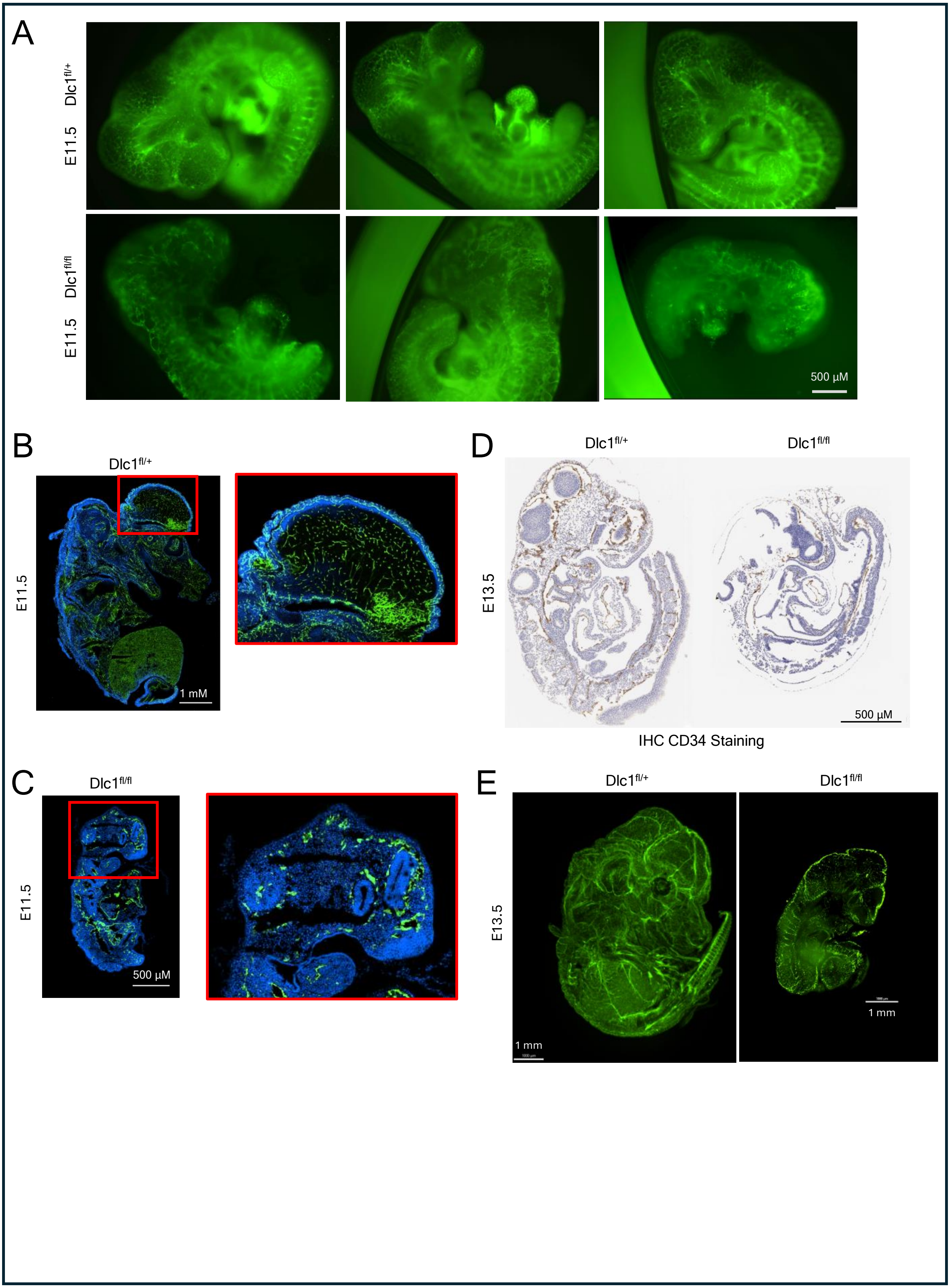
A
E11.5 Dlc1fl/+
E11.5 Dlc1fl/fl
500 µM
B
Dlc1fl/+
E11.5
1 mM
C
Dlc1fl/fl
E11.5
500 µM
D
Dlc1fl/+
Dlc1fl/fl
E13.5
500 µM
IHC CD34 Staining
E
Dlc1fl/+
Dlc1fl/fl
E13.5
1 mm
1 mm

Figure S1

Supplementary Fig. 1. Endothelial-specific deletion of *Dlc1* is embryonically lethal.

(A) Whole-mount imaging of E11.5 embryos with the indicated genotypes through an inverted fluorescent microscope. Cdh5-Cre^{+}/$Dlc1^{fl/fl}$ embryos exhibit reduced size and disrupted vascular architecture compared to control littermates. The green fluorescence is from the ZsGreen reporter.

(B-C) Representative cryosections of E11.5 embryos from Cdh5-Cre^{+}/$Dlc1^{fl/+}$ (B) and Cdh5-Cre^{+}/$Dlc1^{fl/fl}$ (C) as determined by genotyping. Green fluorescence marks Cre-mediated recombination in endothelial cells; blue fluorescence (DAPI) identifies cell nuclei. The red boxes (left) identify the magnified areas (right) of the embryonic brain. Compared to control (B), the $Dlc1^{EC\text{-}KO}$ embryo (C) shows a disrupted vascular architecture and reduced green fluorescence signal in the developing brain.

(D) Immunohistochemical (IHC) detection of CD34 reveals a reduced or disorganized endothelial cell structures in Cre^{+}/$Dlc1^{fl/fl}$ embryos (E13.5) compared to Cre^{+}/$Dlc1^{fl/+}$ littermates.

(E) Whole-mount confocal imaging of E13.5 embryos with the indicated genotypes. Cdh5-Cre^{+}/$Dlc1^{fl/fl}$ embryos exhibit reduced size and degradation compared to control littermates.

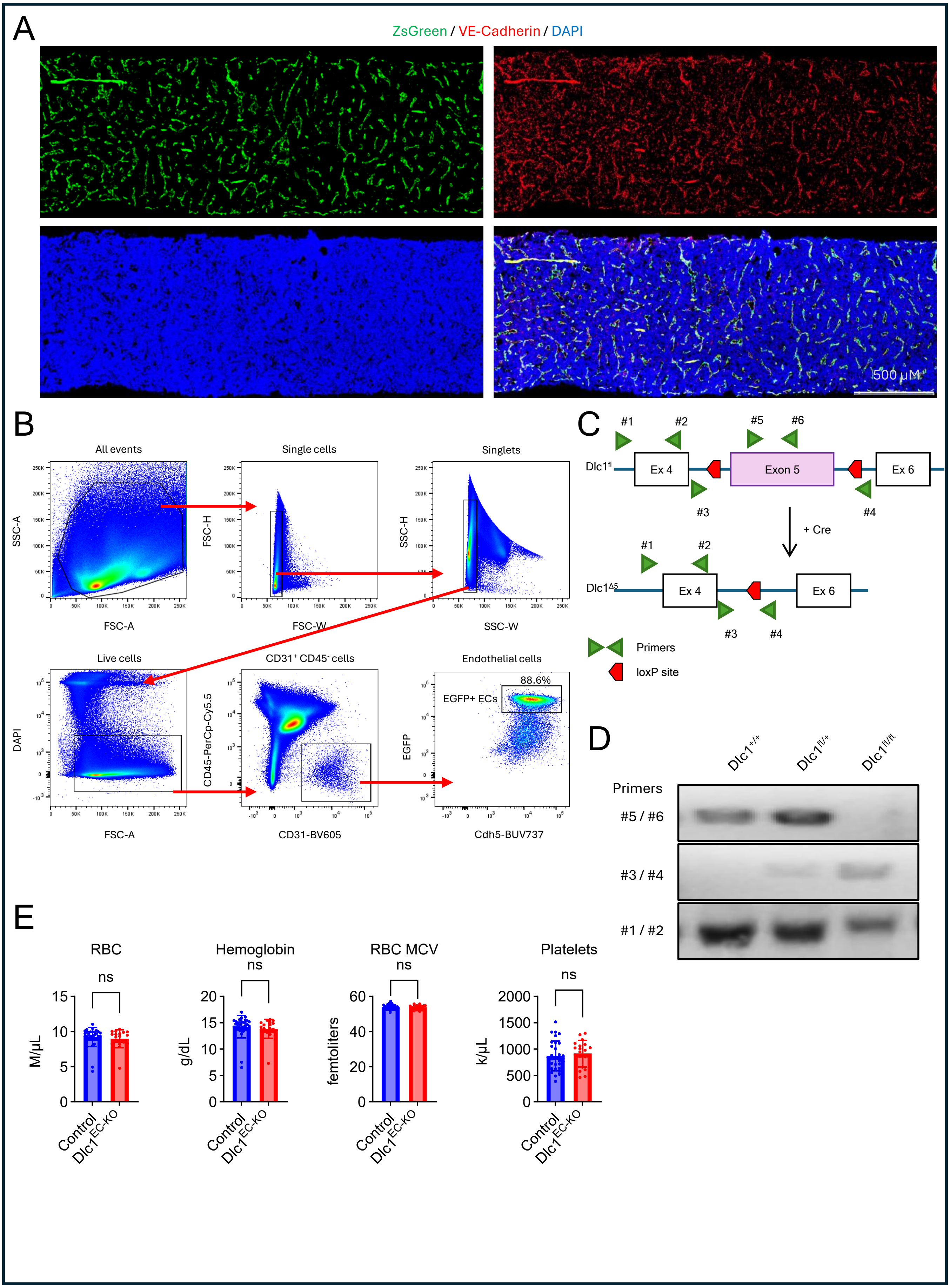
A
ZsGreen / VE-Cadherin / DAPI
500 μM
B
All events
Single cells
Singlets
SSC-A
FSC-A
FSC-H
FSC-W
SSC-H
SSC-W
Live cells
CD31+ CD45- cells
Endothelial cells
DAPI
CD45-PerCp-Cy5.5
CD31-BV605
88.6%
EGFP+ ECs
EGFP
Cdh5-BUV737
C
#1
#2
#5
#6
Dlc1fl
Ex 4
Exon 5
Ex 6
#3
#4
+ Cre
Dlc1Δ5
Primers
loxP site
D
Dlc1+/+
Dlc1fl/+
Dlc1fl/fl
Primers
#5 / #6
#3 / #4
#1 / #2
E
RBC
ns
M/μL
Hemoglobin
ns
g/dL
RBC MCV
ns
femtoliters
Platelets
ns
k/μL
Control
Dlc1EC-KO

Figure S2

Supplementary Fig. 2. Tamoxifen administration efficiently labels and deletes *Dlc1* in adult bone marrow endothelial cells.

(A) Representative confocal images of femoral bone marrow cryosections showing co-staining of VE-Cadherin (red), ZsGreen (green, Cre-reporter), and DAPI (blue), indicating robust labeling of endothelial cells following tamoxifen treatment.

(B) Flow cytometry gating strategy for identifying and isolating bone marrow endothelial cells from *Cdh5-Cre*^{ERT2}/*mTmG* mice. Live, single $CD45^{-}CD31^{+}$ cells were used to identify ECs, followed by selection of VE-Cadherin^{+} EGFP^{+} reporter-positive cells. VE-Cadherin was identified by the Cdh5-BUV737 antibody.

(C) Schematic of primer design for detecting deletion of *Dlc1* exon 5 by genomic PCR. Primers #1 and #2 targeting intron 4 and exon 4 serve as positive control. Primers #5 and #6 target exon 5. The primer pair #3 and #4 spans a >2,500 bp region in the intact (non-recombined) allele, which cannot be amplified under standard PCR extension conditions (20 seconds per cycle). Only the shorter recombined fragment (indicative of exon 5 deletion) yields a detectable product with primers #3 and #4.

(D) Genomic PCR results from sorted endothelial cells of *Dlc1*$^{+/+}$, *Dlc1*$^{fl/+}$, and *Dlc1*$^{fl/fl}$ mice. PCR products using the #3 and #4 primers are only detected in knockout samples, confirming the successful Cre-mediated excision in EGFP^{+} bone marrow endothelial cells.

(E) Red blood cell count (RBC), hemoglobin, red blood cells mean corpuscular volume (RBC MCV), and platelet counts from complete blood count (CBC) analysis of peripheral blood from control and *Dlc1*$^{EC\text{-}KO}$ mice; ns, not significant, unpaired two-tailed Student's t-test. Each dot represents the result from one mouse (Control, n = 31; Dlc1$^{EC\text{-}KO}$, n=19).

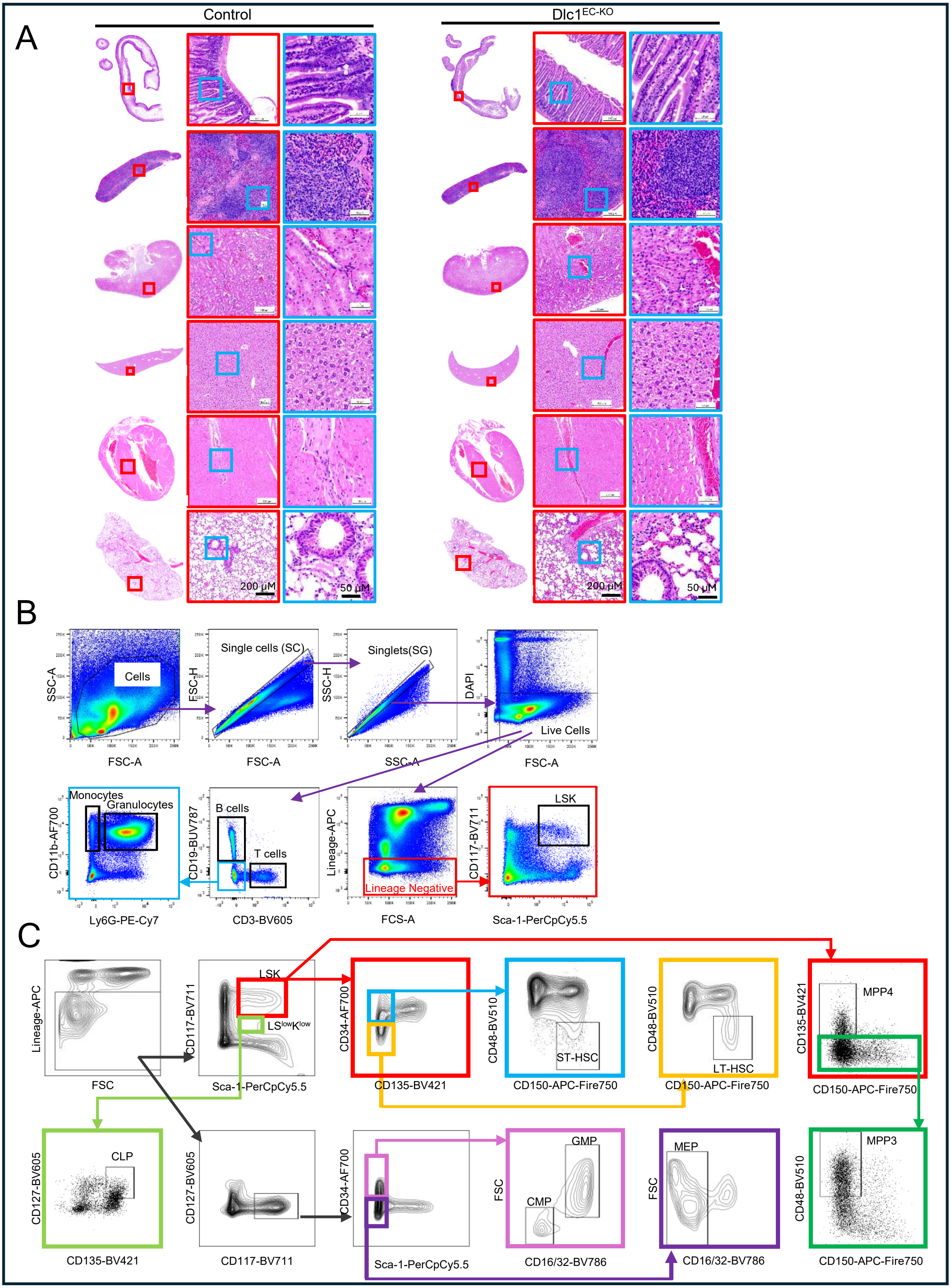

A
Control
Dlc1EC-KO
200 μM
50 μM
200 μM
50 μM
B
Cells
SSC-A
FSC-A
Single cells (SC)
FSC-H
FSC-A
Singlets(SG)
SSC-H
SSC-A
DAPI
Live Cells
FSC-A
Monocytes
Granulocytes
CD11b-AF700
Ly6G-PE-Cy7
B cells
T cells
CD19-BUV787
CD3-BV605
Lineage-APC
Lineage Negative
FCS-A
LSK
CD117-BV711
Sca-1-PerCpCy5.5
C
Lineage-APC
FSC
LSK
LSlowKlow
CD117-BV711
Sca-1-PerCpCy5.5
CD34-AF700
CD135-BV421
CD48-BV510
ST-HSC
CD150-APC-Fire750
CD48-BV510
LT-HSC
CD150-APC-Fire750
MPP4
CD135-BV421
CD150-APC-Fire750
CLP
CD127-BV605
CD135-BV421
CD127-BV605
CD117-BV711
CD34-AF700
Sca-1-PerCpCy5.5
GMP
CMP
FSC
CD16/32-BV786
MEP
FSC
CD16/32-BV786
MPP3
CD48-BV510
CD150-APC-Fire750

Figure S3

Supplementary Fig. 3. Endothelial-specific deletion of *Dlc1* causes no overt organ defects. Flow cytometry gating strategies for identification of bone marrow cell populations.

(A) Representative H&E-stained sections of organs from control and *Dlc1* $^{EC\text{-}KO}$ mice. No gross morphological differences were observed between the two groups in the intestine, spleen, kidney, liver, heart, and lung.

(B) Flow cytometry gating strategy used to identify key hematopoietic cell populations from bone marrow, including: LSK cells (Lineage^{-} Sca-1^{+} c-Kit^{+}), B cells (CD19^{+}), T cells (CD3^{+}), Granulocytes (CD11b^{+}, Ly6G^{+}) and monocytes (CD11b^{+}, Ly6G^{-}).

(C) Flow cytometry gating strategy used to identify key hematopoietic stem and progenitor cell populations from bone marrow; LT-HSC (Lin^{-} Sca-1^{+} cKit^{+} CD34^{-} CD135^{-} CD150^{+} CD48^{-}), ST-HSC (Lin^{-} Sca-1^{+} c-Kit^{+} CD34^{+} CD135^{-} CD150^{+} CD48^{-}), MPP3 (Lin^{-} Sca-1^{+} cKit^{+} CD135^{-} CD150^{-} CD48^{+}), MPP4 (Lin^{-} Sca-1^{+} cKit^{+} CD135^{+} CD150^{-}), CLP (Lin^{-} Sca-1^{low} c-Kit^{low} CD135^{+} CD127^{+}), CMP (Lin^{-} Sca-1^{-} c-Kit^{+} CD34^{+} CD127^{-} CD16/32^{-}), GMP (Lin^{-} Sca-1^{-} c-Kit^{+} CD34^{+} CD127^{-} CD16/32^{+}), MEP (Lin^{-} Sca-1^{-} c-Kit^{+} CD34^{-} CD127^{-} CD16/32^{-}).

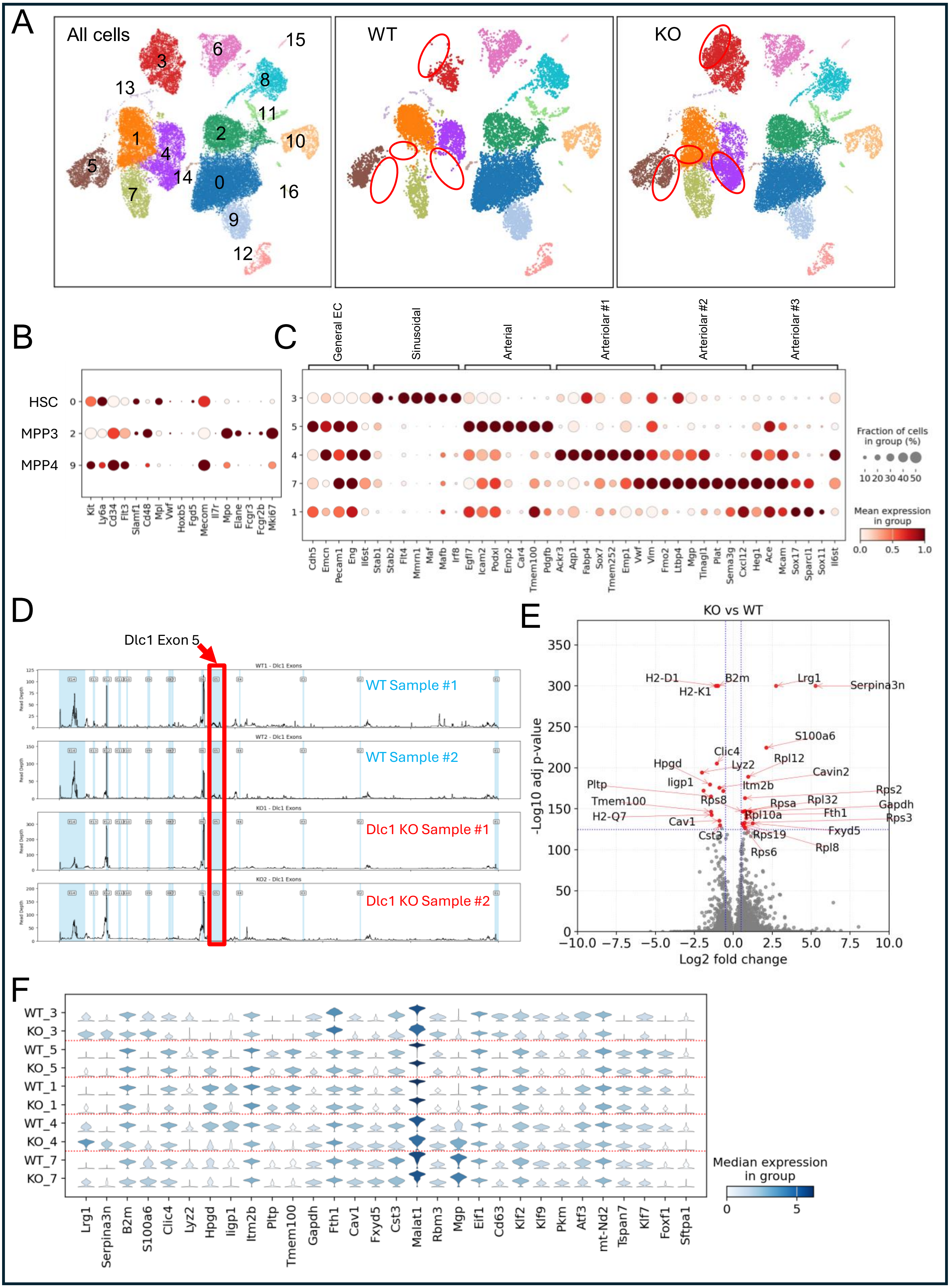
A
All cells
WT
KO
B
HSC
MPP3
MPP4
C
General EC
Sinusoidal
Arterial
Arteriolar #1
Arteriolar #2
Arteriolar #3
Fraction of cells in group (%)
Mean expression in group
D
Dlc1 Exon 5
WT Sample #1
WT Sample #2
Dlc1 KO Sample #1
Dlc1 KO Sample #2
E
KO vs WT
-Log10 adj p-value
Log2 fold change
F
Median expression in group

Figure S4

Supplementary Fig. 4. scRNA-seq reveals altered bone marrow endothelial cell populations in *Dlc1*$^{EC\text{-}KO}$ mice.

(A) UMAP plots showing unsupervised clustering of all (WT and *Dlc1*$^{EC\text{-}KO}$) cells (left), WT controls (middle), and *Dlc1*$^{EC\text{-}KO}$ samples (right). Clusters are numbered 0-16.

(B) Dot plot of marker genes distinguishing hematopoietic stem and progenitor cell (HSPC) subtypes. Cluster 0: HSC; cluster 2: MPP3; and cluster 9: MPP4.

(C) Dot plot of marker genes identifying the endothelial cell subtypes arterial ECs, sinusoidal ECs, and arteriolar ECs.

(D) Read coverage histograms across the *Dlc1* gene locus in WT and *Dlc1*$^{EC\text{-}KO}$ ECs. The red box highlights exon 5, which shows read coverage in WT ECs but nearly no coverage in KO ECs, confirming efficient Cdh5-Cre–mediated excision.

(E) Volcano plot showing differentially expressed genes (DEGs) between Dlc1$^{EC\text{-}KO}$ and WT endothelial cell groups. The top 30 DEGs are indicated. Positive $\log_2$ fold-change values indicate genes upregulated in KO ECs. The $-\log_{10}$ adjusted p-value indicates the significance of differential expression. Dotted vertical and horizontal lines mark the fold-change and significance cutoffs, respectively.

(F) Stacked violin plots showing median expression levels of the 30 selected top DEGs across the EC clusters 3, 5, 1, 4 and 7 from WT and *Dlc1*$^{EC\text{-}KO}$ experimental groups.

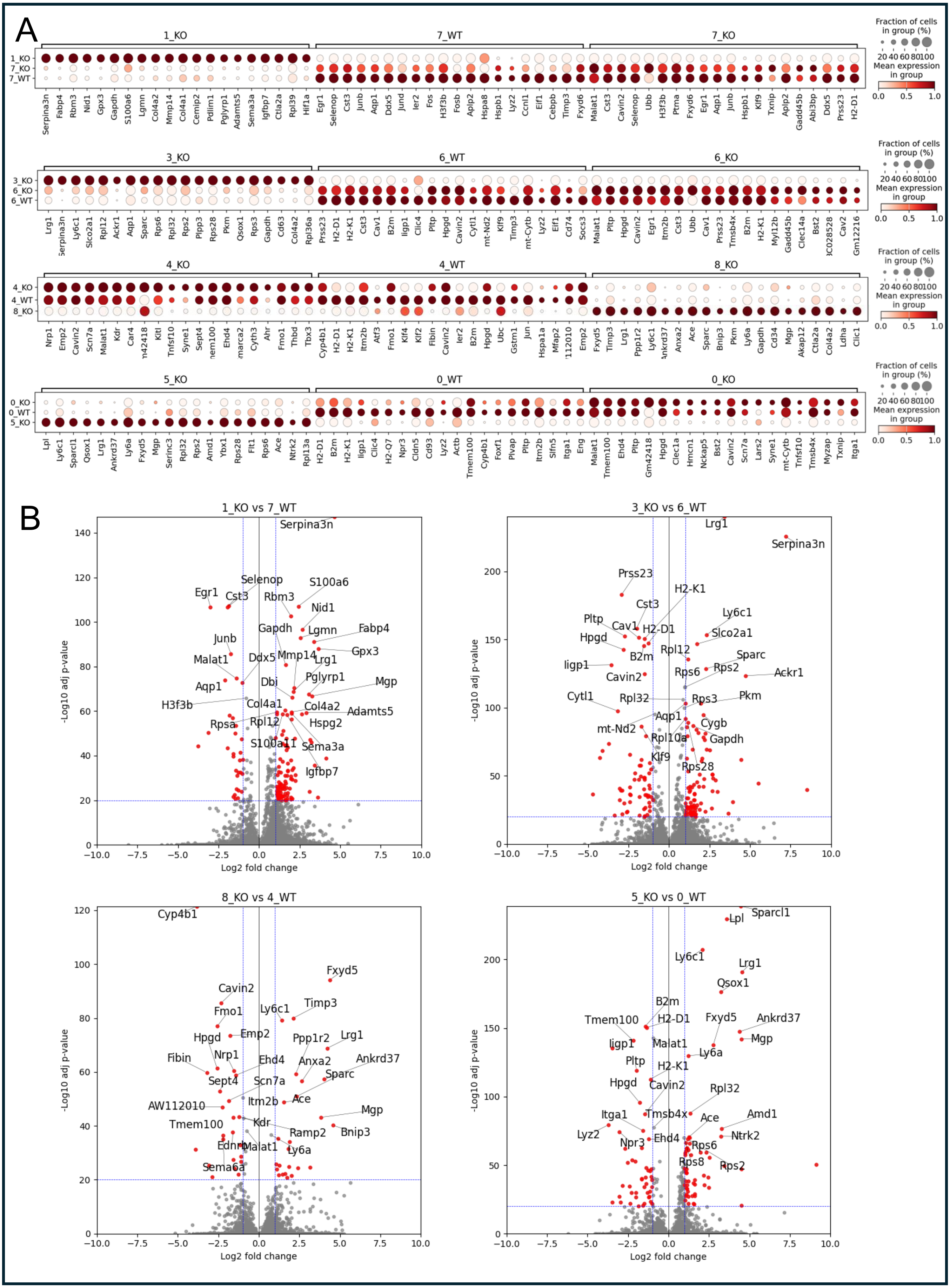
A
1_KO
7_WT
7_KO
1_KO
7_KO
7_WT
Serpina3n Fabp4 Rbm3 Nid1 Gpx3 Gapdh S100a6 Lgmn Col4a2 Mmp14 Col4a1 Cemip2 Pdlim1 Pglyrp1 Adamts5 Sema3a Igfbp7 Ctla2a Rpl39 Hif1a
Egr1 Selenop Cst3 Junb Aqp1 Ddx5 Jund Ier2 Fos H3f3b Fosb Aplp2 Hspa8 Hspb1 Lyz2 Ccnl1 Eif1 Cebpb Timp3 Fxyd6
Malat1 Cst3 Cavin2 Selenop Ubb H3f3b Ptma Fxyd6 Egr1 Aqp1 Junb Hspb1 Klf9 Txnip Aplp2 Gadd45b Abi3bp Ddx5 Prss23 H2-D1
Fraction of cells in group (%)
20 40 60 80 100
Mean expression in group
0.0 0.5 1.0
3_KO
6_WT
6_KO
3_KO
6_KO
6_WT
Lrg1 Serpina3n Ly6c1 Slco2a1 Rpl12 Ackr1 Aqp1 Sparc Rps6 Rpl32 Rps2 Plpp3 Rps28 Pkm Qsox1 Rps3 Gapdh Cd63 Col4a2 Rpl36a
Prss23 H2-D1 H2-K1 Cst3 Cav1 B2m Iigp1 Clic4 Pltp Hpgd Cavin2 Cytl1 mt-Nd2 Klf9 Timp3 mt-Cytb Lyz2 Eif1 Cd74 Socs3
Malat1 Pltp Hpgd Cavin2 Egr1 Itm2b Cst3 Ubb Cav1 Prss23 Tmsb4x B2m H2-K1 Myl12b Gadd45b Clec14a Bst2 Gm12216 Cav2
4_KO
4_WT
8_KO
4_KO
4_WT
8_KO
Nrp1 Emp2 Cavin2 Scn7a Malat1 Kdr Car4 Kitl Tnfsf10 Syne1 Sept4 Ehd4 Cyth3 Ahr Fmo1 Thbd Tbx3
Cyp4b1 H2-D1 H2-K1 Itm2b Atf3 Fmo1 Klf4 Klf2 Fibin Cavin2 Ier2 B2m Hpgd Ubc Gstm1 Jun Hspa1a Mfap2 Emp2
Fxyd5 Timp3 Lrg1 Ppp1r2 Ly6c1 Anxa2 Ace Sparc Bnip3 Pkm Ly6a Gapdh Cd34 Mgp Akap12 Ctla2a Col4a2 Ldha Clic1
5_KO
0_WT
0_KO
0_KO
0_WT
5_KO
Lpl Ly6c1 Sparcl1 Qsox1 Lrg1 Ankrd37 Ly6a Fxyd5 Mgp Serinc3 Rpl32 Rps2 Amd1 Ybx1 Rps28 Flt1 Rps6 Ace Ntrk2 Rpl13a
H2-D1 B2m H2-K1 Iigp1 Clic4 H2-Q7 Npr3 Cldn5 Cd93 Lyz2 Actb Tmem100 Cyp4b1 Foxf1 Plvap Pltp Itm2b Slfn5 Itga1 Eng
Malat1 Tmem100 Ehd4 Pltp Gm42418 Hpgd Clec1a Hmcn1 Nckap5 Bst2 Cavin2 Scn7a Lars2 Syne1 mt-Cytb Tnfsf10 Tmsb4x Myzap Txnip Itga1
B
1_KO vs 7_WT
Serpina3n Selenop S100a6 Egr1 Cst3 Rbm3 Nid1 Gapdh Fabp4 Lgmn Junb Gpx3 Malat1 Ddx5 Mmp14 Lrg1 Pglyrp1 Aqp1 Dbi Mgp H3f3b Col4a1 Col4a2 Adamts5 Rpsa Rpl12 Hspg2 S100a11 Sema3a Igfbp7
3_KO vs 6_WT
Lrg1 Serpina3n Prss23 H2-K1 Cst3 Pltp Ly6c1 Cav1 H2-D1 Slco2a1 Hpgd Rpl12 B2m Sparc Iigp1 Rps2 Ackr1 Cavin2 Rps6 Cytl1 Rpl32 Rps3 Pkm Aqp1 Cygb mt-Nd2 Rpl10a Gapdh Klf9 Rps28
8_KO vs 4_WT
Cyp4b1 Fxyd5 Cavin2 Timp3 Fmo1 Ly6c1 Hpgd Emp2 Ppp1r2 Lrg1 Nrp1 Fibin Ehd4 Anxa2 Ankrd37 Sept4 Scn7a Sparc AW112010 Itm2b Ace Mgp Tmem100 Kdr Ramp2 Bnip3 Ednrb Malat1 Ly6a Sema6a
5_KO vs 0_WT
Sparcl1 Lpl Ly6c1 Lrg1 Qsox1 B2m Tmem100 H2-D1 Fxyd5 Ankrd37 Iigp1 Malat1 Mgp Ly6a Pltp H2-K1 Hpgd Cavin2 Rpl32 Itga1 Tmsb4x Ace Amd1 Lyz2 Npr3 Ehd4 Ntrk2 Rps6 Rps8 Rps2
-Log10 adj p-value
Log2 fold change
−10.0 −7.5 −5.0 −2.5 0.0 2.5 5.0 7.5 10.0

Figure S5

Supplementary Fig. 5. *Dlc1*-depletion defines unique endothelial cell subtypes.

(A) Dot plots showing marker gene expression profiles of KO-unique endothelial cell subclusters, compared individually with the WT and KO components of the corresponding mixed subclusters. Dot size indicates the proportion of cells expressing each gene, and color intensity represents the mean expression level.

(B) Volcano plots showing differentially expressed genes (DEGs) in KO-unique endothelial cell subclusters (5, 1, 8, 3) and the corresponding WT component in the “mixed” subclusters (0, 7, 4, 6).

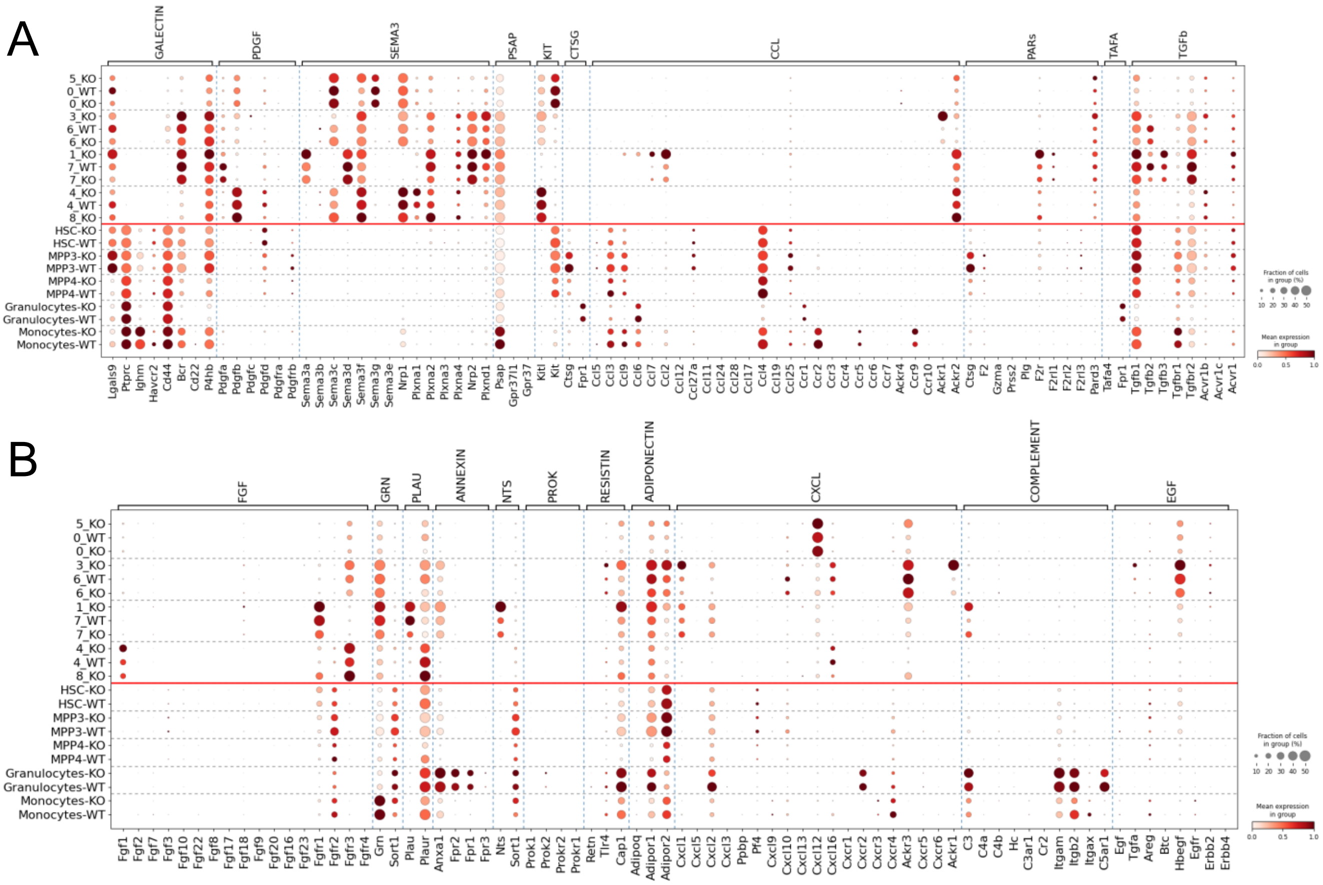
A
GALECTIN
PDGF
SEMA3
PSAP
KIT
CTSG
CCL
PARs
TAFA
TGFb
5_KO
0_WT
0_KO
3_KO
6_WT
6_KO
1_KO
7_WT
7_KO
4_KO
4_WT
8_KO
HSC-KO
HSC-WT
MPP3-KO
MPP3-WT
MPP4-KO
MPP4-WT
Granulocytes-KO
Granulocytes-WT
Monocytes-KO
Monocytes-WT
Lgals9 Ptprc Ighm Havcr2 Cd44 Bcr Cd22 P4hb Pdgfa Pdgfb Pdgfc Pdgfd Pdgfra Pdgfrb Sema3a Sema3b Sema3c Sema3d Sema3f Sema3g Sema3e Nrp1 Plxna1 Plxna2 Plxna3 Plxna4 Nrp2 Plxnd1 Psap Gpr37l1 Gpr37 Kitl Kit Ctsg Fpr1 Ccl5 Ccl3 Ccl9 Ccl6 Ccl7 Ccl2 Ccl12 Ccl27a Ccl11 Ccl24 Ccl28 Ccl17 Ccl4 Ccl19 Ccl25 Ccr1 Ccr2 Ccr3 Ccr4 Ccr5 Ccr6 Ccr7 Ackr4 Ccr9 Ccr10 Ackr1 Ackr2 Ctsg F2 Gzma Prss2 Plg F2r F2rl1 F2rl2 F2rl3 Pard3 Tafa4 Fpr1 Tgfb1 Tgfb2 Tgfb3 Tgfbr1 Tgfbr2 Acvr1b Acvr1c Acvr1
Fraction of cells in group (%)
10 20 30 40 50
Mean expression in group
0.0 0.5 1.0
B
FGF
GRN
PLAU
ANNEXIN
NTS
PROK
RESISTIN
ADIPONECTIN
CXCL
COMPLEMENT
EGF
5_KO
0_WT
0_KO
3_KO
6_WT
6_KO
1_KO
7_WT
7_KO
4_KO
4_WT
8_KO
HSC-KO
HSC-WT
MPP3-KO
MPP3-WT
MPP4-KO
MPP4-WT
Granulocytes-KO
Granulocytes-WT
Monocytes-KO
Monocytes-WT
Fgf1 Fgf2 Fgf7 Fgf3 Fgf10 Fgf22 Fgf8 Fgf17 Fgf18 Fgf9 Fgf20 Fgf16 Fgf23 Fgfr1 Fgfr2 Fgfr3 Fgfr4 Grn Sort1 Plau Plaur Anxa1 Fpr2 Fpr1 Fpr3 Nts Sort1 Prok1 Prok2 Prokr2 Prokr1 Retn Tlr4 Cap1 Adipoq Adipor1 Adipor2 Cxcl1 Cxcl5 Cxcl2 Cxcl3 Ppbp Pf4 Cxcl9 Cxcl10 Cxcl13 Cxcl12 Cxcl16 Cxcr1 Cxcr2 Cxcr3 Cxcr4 Ackr3 Cxcr5 Cxcr6 Ackr1 C3 C4a C4b Hc C3ar1 Cr2 Itgam Itgb2 Itgax C5ar1 Egf Tgfa Areg Btc Hbegf Egfr Erbb2 Erbb4
Fraction of cells in group (%)
10 20 30 40 50
Mean expression in group
0.0 0.5 1.0

Figure S6

Supplementary Fig. 6. Expression of ligands and receptors in *Dlc1*-deficient and WT bone marrow cells sustaining endothelial-to-hematopoietic cell communication.

(A-B) Dot plots showing expression of representative ligand (endothelial cell source) and receptor (hematopoietic cell source) genes used in CellChat analysis. The results include KO-specific endothelial subclusters, the WT and KO corresponding subclusters, and WT and KO hematopoietic cell populations. Dot size indicates the fraction of cells expressing each gene; color intensity reflects mean expression level.

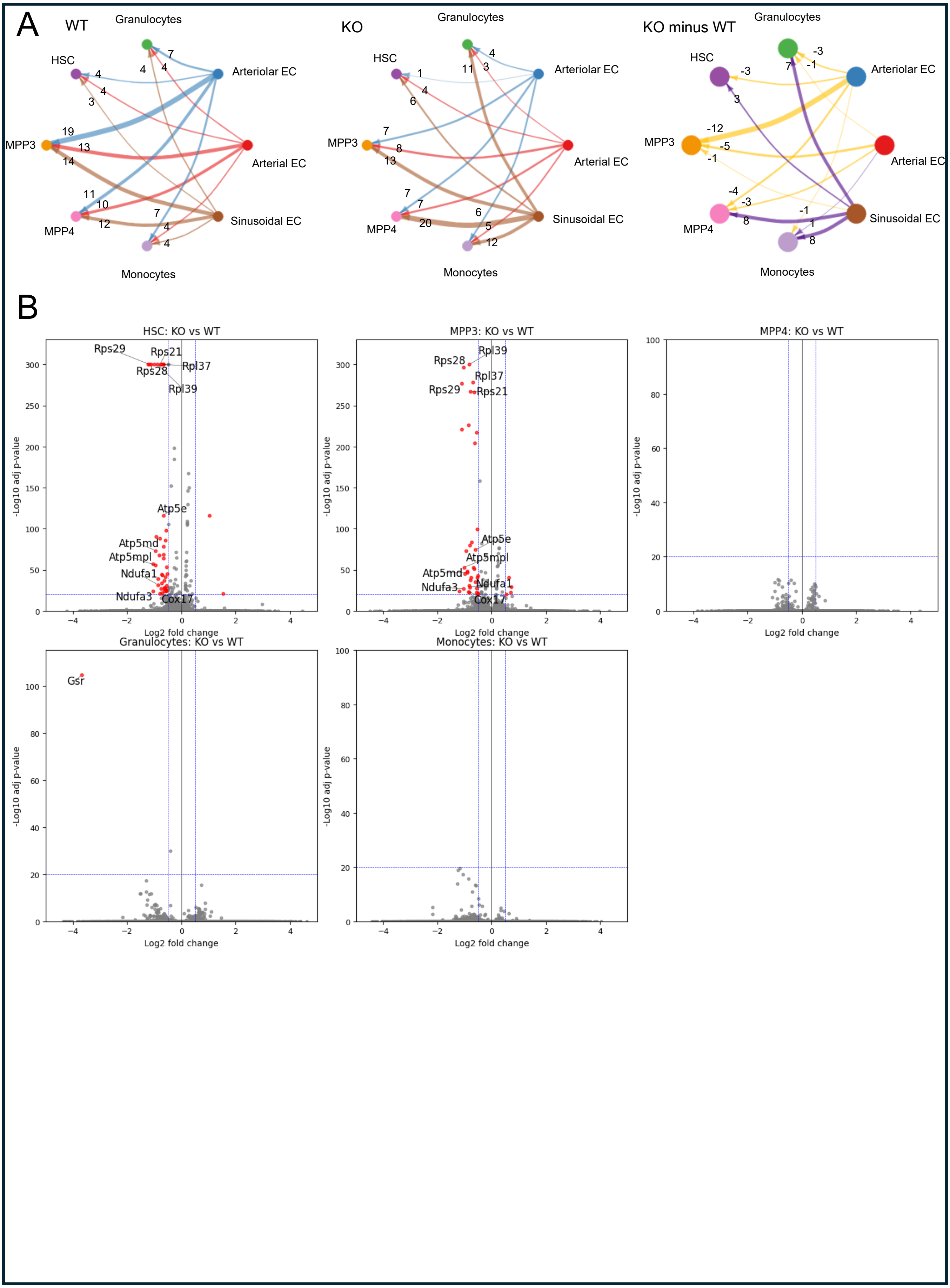
A
WT
KO
KO minus WT
Granulocytes
HSC
Arteriolar EC
MPP3
Arterial EC
MPP4
Sinusoidal EC
Monocytes
B
HSC: KO vs WT
MPP3: KO vs WT
MPP4: KO vs WT
Granulocytes: KO vs WT
Monocytes: KO vs WT
-Log10 adj p-value
Log2 fold change
Rps29
Rps21
Rps28
Rpl37
Rpl39
Atp5e
Atp5md
Atp5mpl
Ndufa1
Ndufa3
Cox17
Gsr

Figure S7

Supplementary Fig. 7. Unique endothelial cell population in *Dlc1* deficient bone marrow exhibit a disrupted communication with hematopoietic cells.

(A) Circular plots showing endothelial-to-hematopoietic communication patterns inferred by CellChat using KO-unique and WT endothelial subclusters. Left: WT endothelial-WT hematopoietic cell networks (this plot is reproduced here from Fig. 8A, left); middle, $Dlc1^{EC\text{-}KO}$-endothelial (from unique subclusters)-$Dlc1^{EC\text{-}KO}$ hematopoietic cell networks; right, network displaying gained or lost interactions in KO relative to WT (KO minus WT). In the left and middle panels, dots and line-segment coloration indicate the endothelial cell source and communication to hematopoietic cells; line-segment width represents the number of detected signaling interactions. In the right panel, purple line-segments denote an increase, and yellow lines denote a decrease of interactions in KO relative to WT; lines width reflects the magnitude of change.

(B) Volcano plots showing differentially expressed genes (DEGs) across the indicated bone marrow hematopoietic cell populations in $Dlc1^{\text{EC-KO}}$ versus WT groups. Red dots denote genes with a significantly altered expression ($|\log_2$ fold change$| > 1$, adjusted $P < 1\text{e-}20$).
